# Acupuncture-Point Stimulation for Postoperative Pain Control: A Systematic Review and Meta-Analysis of Randomized Controlled Trials

**DOI:** 10.1155/2015/657809

**Published:** 2015-10-12

**Authors:** Xian-Liang Liu, Jing-Yu Tan, Alex Molassiotis, Lorna K. P. Suen, Yan Shi

**Affiliations:** ^1^10th People's Hospital of Tongji University, 301 Yanchang Road, Shanghai 200072, China; ^2^School of Nursing, Midwifery and Paramedicine, Australian Catholic University, 1100 Nudgee Road, Banyo, Brisbane, QLD 4014, Australia; ^3^School of Nursing, Jinggangshan University, 28 Xueyuan Road, Ji'an 343009, China; ^4^School of Nursing, The Hong Kong Polytechnic University, Hung Hom, Kowloon, Hong Kong

## Abstract

The purpose of this study was to evaluate the effectiveness of Acupuncture-point stimulation (APS) in postoperative pain control compared with sham/placebo acupuncture or standard treatments (usual care or no treatment). Only randomized controlled trials (RCTs) were included. Meta-analysis results indicated that APS interventions improved VAS scores significantly and also reduced total morphine consumption. No serious APS-related adverse effects (AEs) were reported. There is Level I evidence for the effectiveness of body points plaster therapy and Level II evidence for body points electroacupuncture (EA), body points acupressure, body points APS for abdominal surgery patients, auricular points seed embedding, manual auricular acupuncture, and auricular EA. We obtained Level III evidence for body points APS in patients who underwent cardiac surgery and cesarean section and for auricular-point stimulation in patients who underwent abdominal surgery. There is insufficient evidence to conclude that APS is an effective postoperative pain therapy in surgical patients, although the evidence does support the conclusion that APS can reduce analgesic requirements without AEs. The best level of evidence was not adequate in most subgroups. Some limitations of this study may have affected the results, possibly leading to an overestimation of APS effects.

## 1. Introduction

Nearly 86% of surgery patients experience moderate to severe postoperative pain [1]. Depending on surgery type, as many as half of these patients go on to experience chronic postoperative pain [2]. Unsatisfactory pain control can limit patients' physical activities, prolong recovery time, and contribute to poor quality of life [3, 4]. Pain may also increase postoperative complications, such as postoperative morbidity, and may extend the length of hospitalization, increasing health care costs [5, 6].

Administration of standard analgesics, which are considered generally safe and effective, remains the primary approach to postoperative pain management [7]. However, systemic analgesic administration can induce some adverse effects (AEs), such as nausea, vomiting, depressive symptoms, pruritus, urinary retention, gastrointestinal motility, and ileus [8, 9]. AEs can impair physical and psychological wellbeing and, more seriously, may result in significant morbidity or even mortality [8–10]. To achieve better postoperative pain relief and reduce the requirement for analgesic medication, various nonpharmacological approaches, including educational intervention, relaxation, and acupuncture-point stimulation (APS), have been employed. APS has been lauded as a promising alternative method for achieving postoperative pain relief [11, 12].

APS is a widely used component of traditional Chinese medicine (TCM) together with full-body and auricular approaches [11]. In addition to the most popular methods of manual acupuncture and acupressure, APS can be achieved using modalities such as electrical acupuncture (EA) or laser stimulation and acupoint massage [12]. According to TCM philosophy, the stimulation of target acupoints along meridians produces positive effects by rebalancing* qi* circulation in the body [13]. However, the existence of the* qi* meridian system, as described in TCM, has never been demonstrated scientifically [13]. Nevertheless, the management of various forms of pain remains a key purported benefit of APS [14, 15].

Many animal experiments and clinical studies have examined the therapeutic effects of APS [16]. Early studies showed that APS provided postoperative pain relief in comparison with control groups [17, 18]. Recently, several small trials [3, 19] demonstrated that APS can relieve pain and reduce analgesic requirements associated with hysterectomy and inguinal surgery. However, Sakurai et al. [20] failed to identify any significant change in pain intensity or morphine requirement in surgical patients undergoing acupressure. A prior systematic review found that acupuncture and related techniques aided postoperative pain control, but the quality of evidence was low due to the quality and quantity of included trials, and no subgroup analysis according to acupuncture type was performed [21]. The results of another systematic review conducted by Usichenko et al. [22] suggested that auricular acupuncture was a promising method of postoperative pain reduction, but the heterogeneity of primary studies precluded data synthesis and the evidence was insufficient to draw a definitive conclusion about the treatment's effectiveness. Following the 2008 publication of these reviews, several clinical trials were conducted to evaluate the efficacy of APS for postoperative pain management, generating new evidence on this topic [23–25].

The present systematic review and meta-analysis was conducted to evaluate the effectiveness of APS for pain control following surgical procedures. Therefore, in this study, current evidence generated by randomized controlled trials (RCTs) on the use of APS interventions for postoperative pain management was reviewed and analyzed. Data from patients receiving APS were compared with those from control groups receiving sham/placebo acupuncture, usual care, or no treatment. Compared with the previous literature, this systematic review and meta-analysis incorporates new evidence not previously synthesized and distinguishes between multiple types of APS for postoperative pain control.

## 2. Methods

### 2.1. Study Selection

**Figure 1:**
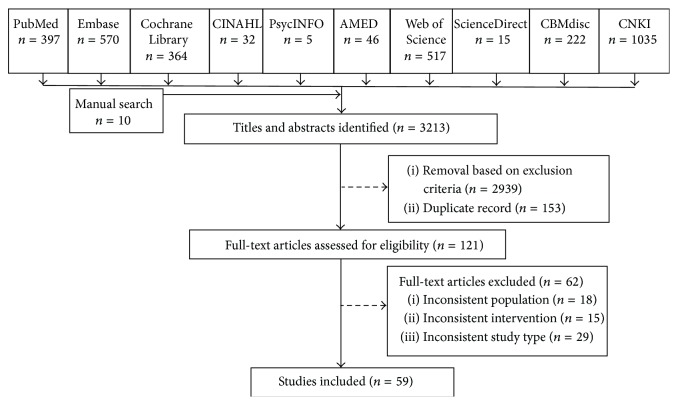
Flow chart of the study selection process. CINAHL, Cumulative Index to Nursing and Allied Health Literature; AMED, Allied and Complementary Medicine Database; CBMdisc, Chinese Biological Medical Literature Database; CNKI, China National Knowledge Infrastructure.

As summarized in Figure 1, we performed a literature search to identify RCTs examining APS interventions in surgical patients with postoperative pain. We searched PubMed, Embase, PsycINFO, Allied and Complementary Medicine, Thomson Reuters Web of Science, ScienceDirect, China National Knowledge Infrastructure, Chinese Biological Medical Literature databases, the Cochrane Central Register of Controlled Trials, and the Cumulative Index to Nursing and Allied Health Literature from inception through 31 May 2014 (search strategies are described in Appendix). Additional relevant articles were identified by screening the reference lists of eligible studies and previous systematic reviews and by performing a manual search for articles published in the last 3 years in eight core TCM journals:* Journal of Beijing University of Traditional Chinese Medicine*,* Journal of Nanjing University of Traditional Chinese Medicine (Natural Sciences)*,* Chinese Journal of Traditional Chinese Medicine and Pharmacy*,* Chinese Journal of Information on Traditional Chinese Medicine*,* Chinese Journal of Basic Medicine in Traditional Chinese Medicine*,* Chinese Journal of Integrated Traditional and Western Medicine*,* Journal of Traditional Chinese Medicine*, and* Journal of Integrative Medicine*. We also used PubMed's “related articles” function to identify additional potentially relevant studies. The electronic search had no language restriction. In the case that there were multiple publications from the same RCT, overlapping results were extracted from one publication.

Two reviewers assessed all potentially relevant articles independently. Disagreements regarding study selection were resolved by discussion, with strict adherence to the inclusion criteria. Studies were selected for inclusion based on the following criteria: (1) RCT; (2) adult (age ≥ 18 years) participants with pain following any surgical procedure; (3) APS intervention (including full-body or auricular manual acupuncture or EA, acupressure, seed embedding, and plaster therapy) conducted by an acupuncturist, TCM practitioner, or other health care providers with qualification and/or training in acupuncture therapy; (4) control group receiving standard treatment (e.g., active pain control approach normally provided to surgical patients, including analgesia medication, nursing guidance, and other usual cares), sham/placebo APS (faked APS intervention), or no treatment (provision of usual postoperative care not involving active analgesic interventions); (5) primary outcome of pain intensity, measured by a valid self-reported instrument such as a visual analog scale (VAS), numerical rating scale (NRS), or verbal reporting; and (6) secondary outcomes of analgesic consumption and APS-related AEs (i.e., any adverse events resulting from APS intervention, minor (e.g., needling site pain), intermediate (e.g., bleeding and hematoma), or serious (pneumothorax and cardiac tamponade)).

In the study selection process, acupuncture was defined as the stimulation of specific acupuncture points along the skin of the body by using thin needles, with or without the application of heat, pressure, or laser light to these same points [12]. EA is similar to acupuncture but involves the use of devices (e.g., a wristwatch-like device and surface electrodes attached to a transcutaneous electrical nerve stimulation device) on acupoint [14]. Seed embedding was defined as an auricular acupressure process involving the embedding of magnetic beads or other seeds within skin-colored adhesive tape, which is placed on the auricular acupoints and retained in situ for several days [6]. In this systematic review, plaster therapy mainly referred to the use of capsicum plaster as an alternative to acupuncture [23].

### 2.2. Quality Assessment

Two reviewers conducted independent assessments of the methodological quality and risk of bias of each RCT using Cochrane Collaboration's risk of bias tool [26]. This tool provides for the assessment of seven domains: sequence generation, allocation concealment, blinding of participants and personnel, blinding of outcome assessment, incomplete outcome data, selective outcome reporting, and “other issues.” Items were scored as showing low, high, or unclear risk of bias [26]. All disagreements on scoring were resolved by discussion. When a sufficient number of studies were available and a meaningful assessment of publication bias could be carried out, a funnel plot was constructed.

Adequate allocation concealment and blinding of outcome assessors were designated as key domains for this assessment, where key domains are not only more likely to influence bias magnitude and direction but also more likely to impact study results. Domain-based evaluation was employed as described in the Cochrane Handbook for Systematic Reviews of Interventions 5.0.2 (updated September 2009). The overall risk of bias was categorized as follows. An overall low risk of bias (plausible bias unlikely to alter the results) was identified when all key domains were assessed as having a low risk of bias. An overall unclear risk of bias (plausible bias that raises some doubt about the results) was identified when one or more key domains were assessed as having an unclear risk of bias. An overall high risk of bias (plausible bias that seriously weakens confidence in the results) was identified when one or more key domains were assessed as having a high risk of bias. Small studies have been shown to overestimate treatment effects and to be at increased risk of bias, allowing critical criteria such as blinding to be compromised. Studies were considered to be at low risk of bias if they had at least 200 participants, at unknown risk if they had 50 to 200 participants, and at high risk if they had fewer than 50 participants.

### 2.3. Data Extraction and Management

Two reviewers independently extracted the following data from included studies using a predefined form: authors, study design, setting, population and participant demographics, intervention characteristics (e.g., acupuncture type, acupoints used, and treatment duration), comparators, outcome measures and instruments, follow-up, and some numeric data (mainly the results of pain intensity and analgesic consumption). We contacted RCT authors by email to obtain data necessary for effect size estimation when such data were missing from publications (e.g., due to aggregated data reporting). When authors did not reply, outcome data presented only in figures and/or graphs were extracted when possible; these data were included in the analysis only when the two reviewers independently obtained the same results.

When a study reported multiple group comparisons (e.g., high electrical stimulation versus low electrical stimulation or usual care and preoperative acupuncture versus postoperative acupuncture or usual care), only data from the treatment group that received the more intensive and comprehensive postoperative intervention were included in the analysis. These data were compared with those from the control group.

### 2.4. Subgroup Analysis

When data were sufficient, subgroup analyses of different types of APS, surgery, and control groups were conducted. Analyses of APS type compared the use of acupoints on the body (EA, manual acupuncture, acupressure, and plaster therapy) and/or auricular points (EA, manual acupuncture, and seed embedding). A subgroup analysis of EA studies compared the use of different devices (e.g., a wristwatch-like device and surface electrodes attached to a transcutaneous electrical nerve stimulation device). Analyses of control group types compared APS with standard treatment (usual care and no treatment) or placebo/sham therapies. On the basis of whether body acupoints, auricular points, or integrative acupoints were stimulated, we also undertook subgroup analyses of surgery types, including abdominal, knee, oral, and cardiac surgeries.

### 2.5. Statistical Analysis

Meta-analysis was performed using Review Manager software (ver. 5.1; available from http://www.cochrane.org/). For continuous outcomes, mean differences with 95% confidence intervals (CIs) were calculated as appropriate. When the same continuous outcome was assessed using different instruments, the standardized mean difference was calculated. For dichotomous outcomes, effect size variables, such as the relative risk (RR), were calculated. In the presence of significant heterogeneity (*χ* ^2^ test, *P* < 0.1), random-effects model was used. Otherwise, fixed-effects model was applied. Descriptive analysis was used when data could not be converted or pooled.

Potential sources of heterogeneity in the outcomes examined are differences in the tool used to measure pain, population differences (e.g., surgery type, age, and sex), and differences in the comparator used (e.g., sham/placebo acupuncture, usual care, and no treatment). We assessed heterogeneity using the *I* ^2^ statistic, which describes the percentage of total variation across trials (low, 0–40%; moderate, 30–60%; substantial, 50–90%; considerable, 75–100%; Chapter 9: Analysing Data and Undertaking Meta-Analyses; The Cochrane Collaboration 2011, available from http://www.cochrane-handbook.org/). To assess which RCTs affected the overall results, sensitivity analyses were performed for the entire sample and subgroups with significant heterogeneity. If heterogeneity was considerable, even with the random-effects model, best evidence synthesis was also used. The evidence was synthesized based on each subgroup. We employed a qualitative modified approach to grading of evidence, as summarized in Table 1.

## 3. Results

### 3.1. Characteristics of Included Trials

The database search yielded 3,203 publications. Manual searching of the reference lists and journals resulted in the retrieval of 10 additional citations. A total of 121 full-text articles were reviewed. After application of the inclusion criteria, 59 RCTs [3, 6, 14, 19, 20, 23–25, 28–78] conducted between 1986 and 2014 were included in the review (Figure 1; Tables 2–4). Nine publications were in Chinese, and the remaining 50 publications were in English. The included studies were conducted in mainland China, Hong Kong, Taiwan, the United States, Germany, Austria, South Korea, Japan, Iran, the United Kingdom, Brazil, Sweden, Singapore, Italy, and Turkey. Sixteen of these RCTs had three arms and three trials had four arms.

RCTs included in the analysis involved a total of 4,578 randomized patients, 4,402 of whom completed the respective studies (APS groups, *n* = 2,097; control groups, *n* = 2,305; 96.16% completion rate). The average sample size was 73 (range, 18–150). Standardized anesthetic and postoperative analgesia regimens were used in all studies. Follow-up duration ranged widely from 7 days [60, 61] to 4 months [41]. Twenty-one RCTs stated that an intention-to-treat analysis was used.

Five types of APS were used: low- and/or high-intensity EA, manual needle acupuncture, seed embedding, acupressure, and plaster therapy. Preoperative and postoperative APS were used in two RCTs, and sham/placebo control was used in 36 studies. Chinese herbs were used as a control in a single study.

Acupuncture points on the body and/or auricular points were stimulated. Commonly used body points included Hegu (LI4), Sanyinjiao (SP6), Zusanli (ST36), and Nei guan (P6); commonly used auricular points included Shen Men (TF4), Stomach (CO4), and Lung (CO14).

### 3.2. Methodological Quality and Risk of Bias of Included Trials

**Figure 2:**
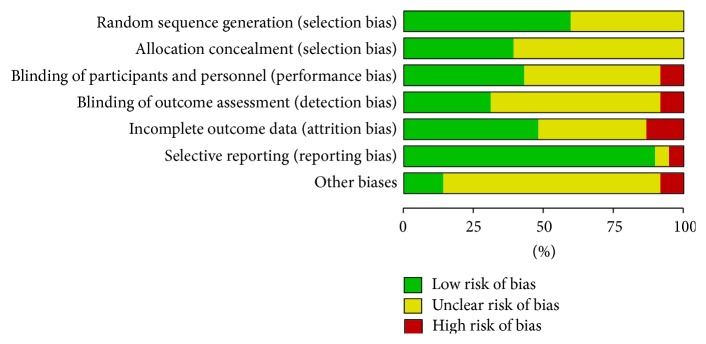
Methodological quality of included studies. Each methodological quality item was qualitatively assessed and is presented as a percentage across all included studies.

**Figure 3:**
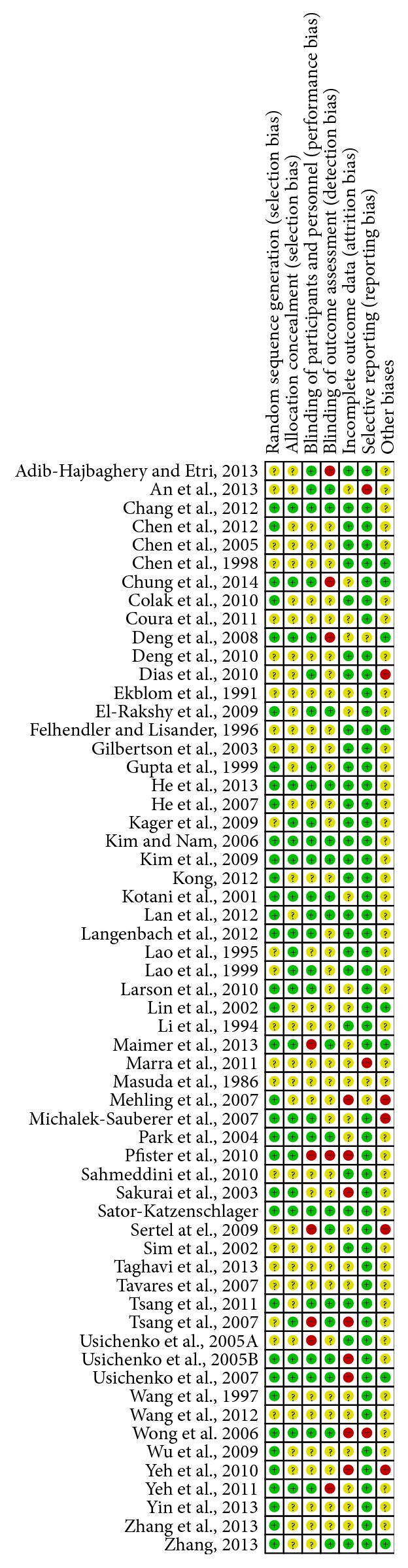
Risk of bias in the included studies. Each bias item was qualitatively assessed.

**Figure 4:**
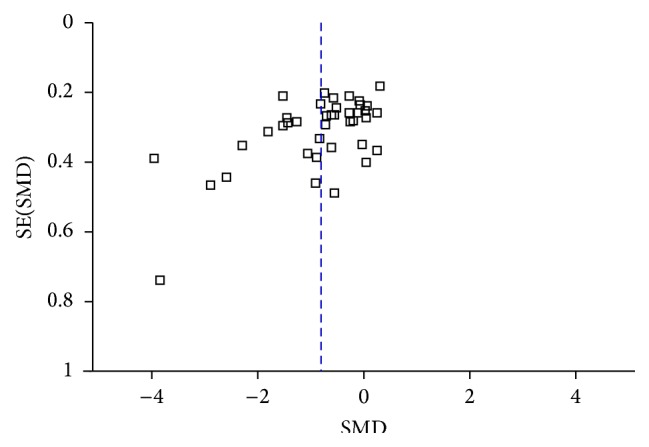
Funnel plot.

The methodological quality of included studies is characterized in Figures 2 and 3. Eighteen (30.5%) publications specifically stated that the outcome assessor was blinded and 23 (38.98%) studies used adequate allocation concealment together with full methodological description. Twelve (20.33%) studies were rated highly in both of these domains and were deemed to be at low risk of overall bias. Forty-two studies (71.19%) were deemed to be at unclear risk of overall bias. Finally, five studies (8.5%) were deemed to be at high overall risk of bias. Allocation concealment was not reported or was described poorly in 24 (40.67%) studies. According to the number of participants, no studies were considered to be at low risk of bias (≥200 participants), 42 studies (71.19%) were at unknown risk of bias (50–200 participants), and 17 studies (28.81%) were at high risk of bias (<50 participants). Visual inspection of funnel plots revealed some substantial asymmetry in comparisons (Figure 4).

### 3.3. Meta-Analysis and Descriptive Analysis of Outcomes

#### 3.3.1. Postoperative Pain

The results of the meta-analysis are reported in Table 5. The RCTs evaluated the efficacy of APS for postoperative pain relief by using VAS scores (*n* = 45), NRS scores (*n* = 5) [53, 66, 74–76], Brief Pain Inventory (BPI) scores (*n* = 2) [34, 75], and a four-point scale and pain-free time to evaluate postoperative pain (*n* = 3) [58, 60, 61]. Two of the five RCTs [53, 74] that used NRS scores reported a significant difference between groups, but these data could not be included in the meta-analysis due to clinical and statistical heterogeneity. One of two RCTs [34, 75] that used BPI scores reported a significant difference between the intervention and control groups, but these data could not be included in the meta-analysis due to different data modes. Three trials [58, 60, 61] using a four-point scale reported that the intervention reduced pain intensity, but one of these studies [58] reported that total or partial pain relief did not differ significantly between the groups. Two trials [60, 61] reported that the APS intervention increased the duration of postoperative pain-free status compared with that of the control groups.

Thirty-eight RCTs used body points for stimulation. Subgroup analyses according to control treatment and meta-analysis of 20 RCTs indicated that APS interventions improved VAS scores significantly in comparison with standard treatment and sham/placebo control (both *P* < 0.00001; Table 5). Similarly, pooled results from 24 trials showed that body APS significantly improved VAS scores in comparison with all control groups (*P* < 0.00001), and subgroup analyses revealed similar improvement compared with standard treatment (*P* < 0.00001) and sham/placebo control (*P* < 0.0001; Table 5). The evidence for body points APS reducing postoperative pain intensity in surgery patients was determined to be of Level I quality based on six overall high quality RCTs [14, 23, 47, 49, 52, 58]. A meta-analysis of pooled results and subgroup analyses of body EA, as well as invasive and noninvasive forms of this treatment, yielded similar results (Table 5). The evidence for body points EA reducing postoperative pain intensity in surgery patients was determined to be of Level II quality based on one overall high quality RCT [14]. High-frequency EA was found to be more effective than low-frequency EA [42, 45]. Pooled results from three RCTs examining acupressure [48, 50, 51] showed a significant difference in VAS scores between intervention and control groups (*P* = 0.01; Table 5), although a fourth study [20] not included in the meta-analysis showed no such difference. The evidence for body points acupressure reducing postoperative pain intensity in surgery patients was determined to be of Level II quality based on three moderate quality RCTs [20, 48, 50] and one low quality RCT [51]. Synthesis of data from two RCTs [23, 47] examining plaster therapy showed a significant reduction in pain intensity compared with standard treatment (*P* < 0.00001) and sham controls (*P* < 0.0001; Table 5), and one other study [49] examining this treatment obtained similar results. The evidence for body points plaster therapy reducing postoperative pain intensity in surgery patients was determined to be of Level I quality based on three overall high quality RCTs [23, 47, 49].

In contrast, meta-analysis including three studies [55–57] revealed no significant effect of manual acupuncture on VAS score. Four [55, 57, 59, 60] of 10 RCTs examining manual acupuncture reported no difference in pain score between the intervention and control groups, whereas the remaining six studies found that this treatment reduced postoperative pain intensity (*P* < 0.05).

Twelve RCTs used body point stimulation for patients with abdominal surgery. The pooled results from eight trials [29, 35, 40, 42, 43, 45, 47, 51] showed that body APS significantly improved VAS scores in these patients (*P* = 0.0006). The evidence for body points APS reducing postoperative pain intensity in patients who had undergone abdominal surgery was determined to be of Level II quality based on one overall high quality RCT [47]. Five [31, 32, 50, 57, 59] RCTs used body point stimulation for patients with knee surgery. Pooled results from four trials [31, 32, 50, 57] showed that body APS did not significantly improve VAS scores for these patients (*P* = 0.16). Each of two RCTs used body point stimulation for patients with oral surgery [60, 61], cardiac surgery [33, 37], hemorrhoid operation [30, 53], or cesarean section [48, 55]. Pooled results from two trials showed that body APS significantly improved VAS scores for patients undergoing cardiac surgery [33, 37] (*P* = 0.002) or cesarean section [48, 55] (*P* < 0.00001). The evidence for body points APS reducing pain intensity in patients who underwent cardiac surgery and cesarean section surgery was determined to be of Level III quality based on two moderate quality RCTs [33, 37]. Other studies could not be included in the meta-analyses due to insufficient data and the different types of surgery.

Fourteen [6, 19, 24, 62–72] RCTs used auricular points for stimulation. Data synthesis from 12 studies showed significantly lower VAS scores in intervention groups than in all types of control group (*P* = 0.001), and similar results were obtained in comparison with standard treatment (*P* = 0.04) and sham/placebo control (*P* = 0.02) groups (Table 5). The evidence for auricular points APS reducing postoperative pain intensity was determined to be of Level I quality based on six overall high quality RCTs [6, 24, 68, 69, 71]. Meta-analysis of data from five studies [6, 24, 62, 64, 65] examining seed embedding also showed a significant effect on VAS score in comparison with all control groups (*P* = 0.02; Table 5). The evidence for auricular points seed embedding reducing postoperative pain intensity was determined to be of Level II quality based on two overall high quality RCTs [6, 24]. Two studies [6, 65] of this auricular APS technique found a gradual reduction in pain, but no significant difference, according to VAS and Short-Form McGill Pain Questionnaire scores. One study [63] of manual auricular acupuncture data reported a significant difference in VAS score, and another study [72] showed a positive trend toward less pain in the intervention group, but meta-analysis of data from four studies [63, 68, 70, 71] showed that this auricular APS type was not associated with significant pain reduction. The evidence for manual auricular acupuncture reducing postoperative pain intensity in surgery patients was determined to be of Level II quality based on two overall high quality RCTs [68, 71]. Meta-analysis of auricular EA data from three studies [19, 66, 69] showed a significant reduction in VAS scores (including those reflecting pain at rest and on huffing and coughing; *P* < 0.0001), although two of the four RCTs examining this treatment found no significant difference due to low pain intensity in intervention groups. The evidence for auricular EA reducing postoperative pain intensity in surgery patients was determined to be of Level II quality based on one overall high quality RCT [69].

Five [6, 24, 63, 68, 70] RCTs used auricular point stimulation for patients with knee surgery. Pooled results from five trials [6, 24, 63, 68, 70] showed that auricular point APS did not significantly improve VAS scores for these patients (*P* = 0.20). Two [19, 62] RCTs used auricular point stimulation for patients with abdominal surgery. Pooled results from both trials [19, 62] showed that auricular point APS significantly improved VAS scores for these patients (*P* = 0.01). The evidence for auricular point stimulation reducing postoperative pain intensity in abdominal surgery patients was determined to be of Level III quality based on two moderate quality RCTs [19, 62].

Seven RCTs [25, 73–78] used integrative APS (combined stimulation of body and auricular points) and evaluated postoperative pain relief using VAS (*n* = 4) and NRS (*n* = 3) scores. This meta-analysis showed a significant effect of integrative APS on pain intensity based on pooled VAS and NRS scores (*P* = 0.03; Table 5) [73–78]. The evidence for integrative APS reducing postoperative pain in surgery patients was determined to be of Level II quality based on five moderate quality [25, 73, 76–78] and two low quality [74, 75] RCTs. Two [77, 78] RCTs used integrative APS for patients with oral surgery. Pooled results from both trials [77, 78] showed that integrative APS did not significantly improve the VAS scores for these patients (*P* = 0.34).

#### 3.3.2. Analgesic Requirement

Forty-three RCTs measured analgesic use, and most studies documented a lesser analgesic requirement in APS intervention groups than in control groups. Meta-analysis of data from six RCTs [3, 25, 34, 42, 43, 55] showed a significant reduction in total morphine consumption in intervention groups compared to the control groups (*P* = 0.0001). Similar results were obtained in the comparison of intervention and sham/placebo control groups (*P* < 0.00001; Table 5). In addition, Lin et al. [42] reported that the morphine requirement after high-frequency EA was decreased by 31% compared with that after low-frequency EA. The evidence for APS reducing analgesic requirement in surgery patients was determined to be of Level I quality based on multiple overall high quality RCTs.

#### 3.3.3. AEs

No serious AEs were associated with APS, and patients were reported to tolerate the intervention well in the 21 RCTs that reported on this outcome. Reported minor AEs included temporary increased pain [74], localized pain or discomfort at insertion sites [60, 71], minor bruising or bleeding [74], constitutional symptoms [74], and a mild burning sensation with erythema [23, 47, 49]. Michalek-Sauberer et al. [67] stated that 38% of patients reported minimal side effects of acupuncture, most commonly fatigue (16%) and ear pain (10%).

### 3.4. Sensitivity and Heterogeneity

Given the detection of obvious heterogeneity (*I* ^2^ > 50%) in meta-analyses, we conducted a sensitivity analysis to remove studies with a greater risk of bias. The results are presented in Table 6. *I* ^2^ values were decreased substantially by the removal of such trials in most comparisons.

## 4. Discussion

In this review, it was determined that there is insufficient evidence thus far to conclude that APS is an effective nonpharmacological approach to the reduction of postoperative pain intensity for surgery patients, although the evidence did show a reduced analgesic requirement with no significant adverse effects in surgery patients. The results may have been affected by some limitations of this study, such as the wide variability of interventions and participants, absence of follow-up evaluation in most included trials, and the often mediocre methodological quality of the included studies. These factors contributed to the high heterogeneity of the data, which limits the strength of the evidence. No studies were considered to be at low risk of bias (≥200 participants) based on the number of participants. These factors may have led to overestimations of APS efficacy.

Given the intensity of surgical trauma, postoperative pain is inevitable and it is deemed to be a serious problem. If this pain is not managed effectively, it can contribute to several clinical risks and affect patients' physical and psychological wellbeing; potential effects include emotional distress, infection, increased myocardial oxygen consumption, and prolonged hospitalization. Associated pathological changes can harm organs and lead to abnormal function [31, 33]. Reduction of postoperative pain is therefore essential.

Our meta-analysis of overall effects from 39 trials showed that interventions involving stimulation of body or auricular points significantly reduced postoperative pain, as measured by VAS scores. Data from studies using integrative APS or manual acupuncture showed uncertain outcomes or no significant change. In one of these studies, Deng et al. [75] suggested that these results may be due to the insufficient strength of APS to produce analgesic effects.

Among body APS studies, the largest subgroup analyzed, all intervention types except manual acupuncture significantly reduced postoperative pain. The precise analgesic mechanism of body APS remains unclear. However, it has been found to facilitate central nervous system release of met-enkephalin and dynorphins into the spinal fluid, causing synergistic pain relief with exogenous opioid medication and production of pain-producing substances, such as potassium and lactic acid [31, 34, 39, 79]. The finding that high-frequency EA at body points was more effective than low-frequency EA may be due to differences in opioid peptide release [33].

Similarly, auricular APS therapies were found to significantly reduce postoperative pain, with the exception of manual acupuncture. The most commonly used auricular point is Shen Men, which generates analgesic, sedative, and anti-inflammatory effects [6]. It also increases endorphin secretion and serotonin production, thereby suppressing the transmission of pain messages and thus pain perception [80]. The results for integrative (auricular and body) APS are less clear; this treatment was found to significantly reduce NRS and VAS scores. Thus, the existing evidence neither supports nor refutes the effectiveness of integrative APS for postoperative pain control.

We also undertook subgroup analyses of surgery types, including abdominal, knee, oral, cesarean, and cardiac surgeries. The meta-analysis results showed that body point acupuncture stimulation and auricular therapy had no significant change on VAS scores for patients undergoing knee surgery. The same trend was observed for patients receiving integrative acupoint stimulation and undergoing oral surgery. Short-term APS stimulation may have been insufficient to reduce patients' pain intensity after knee or oral surgery, or the postoperative rehabilitation program may have affected the results of APS interventions [57]. Rigorously designed large-scale RCTs are needed to identify the effects of APS for these kinds of patients.

This analysis also showed that APS significantly reduces patients' postoperative analgesic requirement. Given the dose-response relationship between analgesics and related adverse effects [81], any nonpharmacological method that reduces the use of analgesic medication is likely to be beneficial. Lin et al.'s [42] finding of reduced morphine requirement after high-frequency EA compared with that after low-frequency EA demonstrates the existence of a dose-response relationship in this treatment as well. However, analgesic requirements are controlled by the health care staff and directly affected by the surgery type and patient's economic condition. Thus, analgesic medication use is not a particularly reliable indicator for the effects of APS.

No APS study reported the occurrence of a serious adverse event, although some minor (mild and transient) side effects were reported. To prevent such effects, APS should be carried out by experienced, well-trained health care professionals who understand the theories underlying this therapy and take necessary precautions.

APS may produce strong placebo effects; for example, sham acupuncture did not affect analgesic-related side effects but did exert a moderate pain-relieving effect [42]. The use of sham/placebo control groups, as in 36 of the examined RCTs, enables clear distinction between true and placebo effects. This meta-analysis showed that the true effects of APS were much stronger than placebo effects. Short-term APS and placebo interventions have shown similar effects, but long-term APS treatment causes beneficial changes in specific brain areas [82].

A small sample size can distort the results of meta-analyses, by overestimating treatment effects, probably due to methodological weaknesses [83]. In our review, no studies were considered to be at low risk of bias (≥200 participants) on the basis of sample size. Forty-two studies (71.19%) were at an unknown risk of bias (50–200 participants), and 17 studies (28.81%) were at a high risk of bias (<50 participants).

In this review, statistical heterogeneity was considerable, even with use of the random-effects model. The best level of evidence was not found for most forms of APS, suggesting that there is, thus far, insufficient evidence to conclude that APS is an effective method for reducing pain intensity in postoperative patients. Within the available body of evidence, there is Level I evidence supporting the effectiveness of body points plaster therapy. Additionally, there is Level II evidence supporting the use of body points EA, body points acupressure, and body points APS in abdominal surgery patients specifically, as well as Level II evidence supporting the use of auricular points seed embedding, manual auricular acupuncture, and auricular EA in surgery patients. Meanwhile, there is only Level III evidence for the use of body points APS in patients who have undergone cardiac surgery and a cesarean section and Level III evidence for the use of auricular point stimulation for pain reduction after abdominal surgery. The main reason that better levels of evidence were not achieved was the methodological quality of the included studies, with only 13 (22.03%) studies meeting at least five of the seven Cochrane review criteria and only 12 (20.33%) studies that were rated highly in key domains being considered at low risk of overall bias.

Two systematic reviews [21, 22] with objectives similar to those of the present study were published in 2008, but overall they produced low quality evidence due to the insufficient quality of included trials. A number of the clinical trials included in the present analysis also had some methodological problems that may have affected their efficacy results. However, we examined all types of APS, with combined and separate analyses of body, auricular, and integrative APS. Rigorously designed large-scale RCTs are needed to identify an optimal standard APS program.

### 4.1. Study Limitations

Some limitations of this study may have affected the results. For example, the wide variability in APS and surgery types, populations, intervention durations, and timing of outcome measurement may be the main factors underlying the observed heterogeneity, which limits the strength of the study results. The small samples and absence of follow-up evaluation in most included trials may have led to overestimation of the effects of APS. Methods of randomization, blinding, and allocation concealment were not reported or were poorly described in some trials, making quality assessment difficult. In addition, visual inspection of the funnel plots revealed some substantial asymmetry in comparisons; thus, the possibility of publication bias (i.e., preference for publication of significant over nonsignificant results) cannot be excluded. In addition, the end-points of included studies varied. End-points in Gilbertson et al. [41] and Chen et al. [32] were 4 and 3 months, respectively. When removing these two studies, the *I* ^2^ values were decreased markedly (Table 6). Therefore, the end-points of included studies have important biases in this review. Future studies of APS should be designed rigorously to ensure a high level of methodological quality.

### 4.2. Implications for Practice and Research

The major advantages of APS are related to its clinical safety, favorable effects in postoperative pain relief, and low complication rate following surgery [58]. Clinical nurses and other health care providers should thus be encouraged to learn and implement this simple, convenient, and economical method of postoperative pain control in routine clinical care [34].

Our findings have implications for research on the precise mechanism of APS in postoperative pain relief. Optimal acupoint selection, session duration, stimulation intensity, and application frequency have not been established. A standardized APS program for postoperative pain management should be designed using an evidence-based method. Because available evidence for integrative APS and manual acupuncture is inconclusive, further studies should focus on further assessing the effects of these treatments on postoperative pain control. Moreover, the best APS type for the reduction or elimination of long-term opioid use and the long-term effects of APS therapies remain unknown. Thus, large-scale multicenter RCTs with long-term follow-up periods should be conducted to verify the short- and long-term effects of APS on postoperative pain control. Furthermore, more attention should be paid to the economic effects of APS in health care systems.

In conclusion, this study indicates that, thus far, there is still insufficient evidence to conclude that APS is an effective method for controlling postoperative pain in surgery patients, although the evidence does suggest that APS can reduce patients' analgesic requirement with no significant adverse effects. The best level of evidence was not adequate in most subgroups. Some limitations of this study may have affected the results, leading to an overestimation of the effects of APS. Rigorously designed large-scale RCTs are needed to identify the effects of APS.

## Supplementary Material

Supplementary Material including the search strategies of each database, MeSH terms, key words, and free words such as “acupuncture,” "acupuncture therapy," "acupuncture analgesia," "acupuncture points," "acupressure," "auriculotherapy,” "acupuncture, ear," "perioperative period," "postoperative period," "preoperative period," "intraoperative period," “pain,” “ache,” "chronic pain," "analgesia," were used in the search strategies.

## Figures and Tables

**Table 1:** Qualitative modified approach to grading of evidence.

Level	Description
I	Evidence obtained from multiple relevant high quality randomized controlled trials

II	Evidence obtained from at least one relevant high quality randomized controlled trial or multiple relevant moderate or low quality randomized controlled trials

III	Evidence obtained from at least one relevant moderate or low quality randomized controlled trial with multiple relevant observational studies, or evidence obtained from at least one relevant high quality nonrandomized trial or observational study with multiple moderate or low quality observational studies

IV	Evidence obtained from multiple moderate or low quality relevant observational studies

V	Opinion or consensus of large group of clinicians and/or scientists

Source: [27].

**Table 2:** Characteristics of RCTs examining body acupoint stimulation included in the meta-analysis.

First author, year, setting	Study design	Participants (*n*), age (years)	Surgery type	APS type	Intervention (acupoints, parameters)		Follow-up	Main outcomes
					Intervention group	Sham/control group		
An, 2013, China [28]	Double-blind RCT, three groups	Randomized = 120 Completed = 120 EA: 40, 40.7 (12.1) TEAS: 40, 42.7 (10.9) Control: 40, 39.1 (10.9)	Supratentorial tumor resection	EA	EA/TEAS groups: LI4/TE5, BL63/LV3, ST36/GB40 Duration: throughout operation	Standard treatment	NR	VAS, recovery time, AE, PCA

Yin, 2013, China [29]	RCT	Randomized = 60 Completed = 60 Intervention: 30, 35.1 (8.6) Control: 30, 36.7 (10.3)	Gynecological laparoscopic surgery	EA	ST36 and ST34 Duration: 30 min	General anesthesia	NR	VAS, PONV, exhaust defecation

Taghavi, 2013, Iran [3]	Randomized, double-blind, placebo- controlled study	Randomized = 90, 25 (5) Completed = 90 Intervention: 45 Placebo: 45	Inguinal surgeries	EA	LI4 and SD36 Duration: 20 min sessions 30 min preoperatively, 1 and 2 h postoperatively	No electrical excitement through needles	NR	VAS, PCA, vital signs, side effects

Zhang, 2013, China [30]	RCT	Randomized = 80 Completed = 80 Intervention: 40 (19–64) Control: 40 (20–65)	Hemorrhoidectomy	EA	BL30 Duration: 30 min, twice a day	50 mg tramadol hydrochloride	NR	VAS, edema score, analgesics requirement, AE

Lan, 2012, China [31]	RCT	Randomized = 68 Completed = 60 Intervention: 30, 76 (6) Sham: 30, 75 (5)	Total knee arthroplasty	EA	Bilateral P6, L14; ST36, and GB31 Duration: 30 min before incision; 2, 4, 20, and 44 h postoperatively	Stimulation 30 min before incision at 0 mA	NR	Analgesics, VAS, RSS, AE

Chen, 2012, China [32]	RCT	Randomized = 70 Completed = 70 Intervention: 35, 67.4 (5.1) Control: 35, 65.6 (5.1)	Total knee arthroplasty	EA	SP10, ST34, ST35, EX-LE4, GB34 Duration: 30 min daily for 1-2 postoperative weeks, twice a week in postoperative weeks 3–12	Routine rehabilitation therapy	12 weeks post-op	HSS, ROM, VAS, MMT

Coura, 2011, Brazil [33]	Prospective, randomized, controlled study	Randomized = 22 Completed = 22 Intervention: 13, 56.2 (11.8) Control: 9, 62.5 (10.8)	Cardiac surgery	EA	LI4–LI11, LR3–ST36, and PC6–TE5 acupoints Duration: 30 min	LI4–LI11, LR3–ST36 acupoints; device turned off but connected, 30 min	NR	Analgesics/boluses required, VAS, hospitalization length

Yeh, 2011, Taiwan [34]	Single-blind, randomized, placebo- controlled trial	Randomized = 90 Completed = 90 Intervention: 30, 60.7 (12.0) Sham: 30, 57.2 (15.7) Control: 30, 63.2 (14.0)	Spinal surgery	EA	BL40, GB34, HT7, P6s Duration: 20 min, 3 and 4 h postoperatively	Sham: same acupoints, not meridian acupoints Control: no AES intervention	NR	VAS, BPI, opiate requirement

Deng, 2010, China [35]	RCT	Randomized = 60 Completed = 60 Intervention: 30, 50.2 (11.0) Control: 30, 50.1 (8.5)	Gastrectomy, proctocolectomy	EA	LI4, PC8, ST25, and BL25 acupoints Duration: 30 min	Simple general anesthesia	NR	VAS, satisfaction, somnus score, PCA, BP, HR, SpO_2_, PONV

Sahmeddini, 2010, Iran [36]	Randomized, double-blind study	Randomized = 90 Completed = 90 Intervention: 45, 27 (11) Control: 45, 29 (10)	Septoplasty	EA	LI4, LI11, HT7, HC6 acupoints Duration: 5 min prior to start of surgery	EA system pasted over hand without needle and stimulation.	NR	VAS, pulse rate, arterial pressure, AE

Colak, 2010, Turkey [37]	RCT	Randomized = 30 Completed = 30 Intervention: 15, 52.3 (8.1) Control: 15, 51.5 (8.5)	Coronary surgery	EA	LI4, LI11, ST36, PC6, LIV3 Duration: 20 min after a 24 h rest interval and then daily for the first 7 postoperative days	Pharmacological analgesia	NR	VAS, analgesic intake, pulmonary function, postoperative complications

Larson, 2010, USA [38]	Single-blind RCT	Randomized = 122 Completed = 122 Intervention: 61 Control: 61	Outpatient plastic surgery	EA	P6 Duration: NR	Standardized pharmacological treatment	NR	Nausea, vomiting, pain, medications required, hospitalization length

El-Rakshy, 2009, UK [39]	Randomized, double-blind, comparative study	Randomized = 107 Completed = 102 Intervention: 58 Control: 44	Abdominal hysterectomy, laparoscopic cholecystectomy	EA	Bilateral CV2, GV4, BL32, BL23, LI4, PC6; LR3, SP6, L14, PC6 Duration: during operation	Morphine sulfate via PCA	NR	Morphine requirement, pain, nausea, vomiting

He, 2007, China [40]	RCT	Randomized = 60 Completed = 60 Intervention: 30, 57.65 (6.67) Control: 30, 58.07 (7.02)	Radical surgery for intestinal cancer	EA	“E pang 3 line” and “Ding pang 1 line” acupoints Duration: from 20 min preoperatively to end of operation	Epidural analgesia at end of operation	NR	VAS, BCS, gastrointestinal function

Wong, 2006, Hong Kong [14]	Randomized, double-blind, placebo- controlled trial	Randomized = 27 Completed = 25 Intervention: 13, 64.6 (8.0) Sham: 12, 64.5 (8.5)	Thoracotomy	EA	LI4, GB34, GB36, TE8 acupoints Duration: two 30 min sessions a day for the first 7 postoperative days	Same acupoints, mimic prick sensation without piercing skin	NR	Peak flow rate, chest drain, VAS, PCA, complications

Gilbertson, 2003, USA [41]	RCT	Randomized = 40 Completed = 40 Intervention: 20, 43 (9.195) Sham: 20, 47.81 (6.765)	Arthroscopic acromioplasty	EA	Acupoints chosen with primary expectation of localized benefit to the operated shoulder Duration: postoperative days 3–8 and then three times per week for 1 month (12 sessions)	Similar to true acupoints and needles, connected but not functioning, blinking red light	4 mos.	UCLA, VAS, analgesic use, SF-36, ROM

Lin, 2002, Taiwan [42]	RCT, four groups	Randomized = 100 Completed = 100 M/F = 0/100 Control: 25, 39 (8) Sham: 25, 41 (12) LF EA: 25, 38 (7) HF EA: 25, 42 (13)	Lower abdominal surgery	EA	ST36 acupoint Duration: 20 min before anesthesia induction	Control group: no stimulation Sham group: needle insertion but no electrical stimulation	NR	VAS, analgesic, PCA demand, HR, BP, SpO_2_, opioid-related AE

Sim, 2002, Singapore [43]	RCT, three groups	Randomized = 90 Completed = 90 Control: 30, 47 (6) Preoperative EA: 30, 46 (5) Postoperative EA: 30, 45 (4)	Gynecological lower abdominal surgery	EA	ST36 and PC6 acupoints Duration: 45 min	Control group: 45 min placebo EA	NR	VAS, morphine requirement, AE, satisfaction

Chen, 1998, USA [44]	RCT	Randomized = 100 Completed = 100 Sham TENS: 25, 45 (12) No TENS: 25, 44 (13) Dermatomal TENS: 25, 44 (13) Acupoint TENS: 25, 43 (13)	Total abdominal hysterectomy, myomectomy	EA	Dermatomal TENS: dermatomal levels corresponding to incision. Acupoint TENS: ST36 Duration: every 2-3 h while awake, at bedtime, on waking in morning	Sham: no electrical stimulation No TENS: shoulder electrical stimulation (nonacupoint)	NR	VAS, PCA demand, analgesic, recovery time, AE

Wang, 1997, USA [45]	Prospective, randomized, sham-controlled, single-blind study	Randomized = 101 Completed = 101 PCA only: 26, 44 (10) Sham TAES: 25, 44 (9) LF TAES: 25, 45 (9) HF TAES: 25, 43 (8)	Lower abdominal surgery	EA	LI4 acupoint Duration: every 2 h for 30 min while awake	PCA only Sham TAES	NR	Hospitalization length, VAS, analgesia required, PCA need, AE, recovery time

Masuda, 1986, Japan [46]	Controlled study	Randomized = 24 Completed = 24 Intervention: 11, 48 (13) Control: 13, 49 (10)	Eye surgery	EA	“Gohkoku” (Hoku) and “Shikoh” (Chikkou) acupoints Duration: from 30 min before surgery until end of operation	Usual neuroleptanesthesia without acupuncture	NR	Pain, anesthetics required, swelling circulatory,

Kim, 2009, Korea [23]	Double-blind, placebo- and sham-controlled study	Randomized = 84 Completed = 84 Intervention: 28, 27.7 (9.1) Sham: 28, 28.6 (8.1) Control: 28, 29.2 (9.3)	Orthognathic surgery	Plaster therapy	Bilateral LI4 acupoints Duration: 30 min before anesthesia induction in first operation, 8 h/day for 3 postoperative days	Sham: inactive tape Control: inactive tape at bilateral LI4 points and deltoid regions	NR	VAS, PCA, AE, PONV, satisfaction score

Kim, 2006, Korea [47]	Double-blind, placebo- and sham-controlled study	Randomized = 90 Completed = 90 Intervention: 30, 41.5 (9.7) Sham: 30, 43.2 (10.7) Control: 30, 42.0 (9.2)	Abdominal hysterectomy	Plaster therapy	ST36 acupoint Duration: 30 min preoperatively, 8 h/day for 3 postoperative days	Sham: plaster on sham acupoints Control: inactive tape without plaster	NR	PCA, VAS, PONV, urinary retention, AE, satisfaction

Park, 2004, Korea [49]	Randomized, double-blind, sham-controlled study	Randomized = 150 Completed = 150 Intervention: 50, 43.8 (32–39) Sham: 50, 44.9 (33–61) Control: 50, 42.6 (32–47)	Total abdominal hysterectomy	Plaster therapy	K-A20 acupoint Duration: 8 h beginning 30 min before anesthesia induction	Sham: placebo tape at K-A20; PAS placed on the lateral thighs as in Group K Placebo: placebo tape placed at K-A20 and on lateral thighs as in Group K.	NR	Sore throat, PONV, fentanyl dose, AE

Adib-Hajbaghery, 2013, Iran [51]	Single-blind RCT	Randomized = 70 Completed = 70 Intervention: 35, 26.89 (9.59) Control: 35, 31.17 (14.79)	Appendectomy	Acupressure	LE7 acupoint Duration: 7 h	Sham point opposite LE7	NR	VAS, nausea, vomiting

Chen, 2005, Taiwan [48]	RCT	Randomized = 104 Completed = 104 Intervention: 52, 32.69 (4.09) Control: 52, 32.27 (4.74)	Cesarean section (CS)	Acupressure	P6 acupoint Duration: 20 min per arm before and within 24 h after CS	Postoperative nursing	NR	DDQ, RINV, VASA, STAI, VAS

Sakurai, 2003, Austria [20]	RCT	Randomized = 53 Completed = 53 Intervention: 23, 43 (16) Control: 30, 49 (14)	Abdominal surgery	Acupressure	Nei guan, Zusanli, Sanyinjiao, and Gongsun acupoints Duration: NR	Standard anesthesia monitoring	NR	VAS, PONV, side effects, antiemetic requirement

Felhendler, 1996, Sweden [50]	RCT	Randomized = 40 Completed = 40 Intervention: 20, 32 (22–44) Control: 20, 35 (29–45)	Knee arthroscopy	Acupressure	ST1, ST45, SP1, SP21, SP4, BL1, BL67, KI1, KI27, KI4, GB1, GB44, LR1, LR14, LR5 Duration: 30 min after waking from anesthesia	15 nonacupoints, no active stimulation	NR	VAS, cardiovascular measurements

Maimer, 2013, Germany [52]	Prospective, randomized, controlled, observer-blinded clinical trial	Randomized = 100 Completed = 100 Control acupuncture: 33, 65 (10) Classic Chinese acupuncture: 34, 68 (11) Control: 33, 66 (10)	Heart surgery	Manual acupuncture	Control acupuncture: LI4, SI6, BL60, EX1, GV20, GB8, ST8 Classic acupuncture: P6, ST34, SP10, ST44, K3R, LIV2 Duration: needle stimulation by rotation for ~5 s at insertion, after 10 min, before removal after 20 min	Standard treatment with no additional acupuncture	NR	PPR, FVC

Langenbach, 2012, Germany [53]	Patient-blind RCT, three groups	Randomized = 50 Completed = 50 Intervention: 17, 62 (15) Sham: 16, 48 (17) Control: 17, 57 (16)	Stapled hemorrhoidopexy	Manual acupuncture	DU2, DU20, BI30, BI57, MA44, PE6 Duration: 20–30 min at 4 pm on day of surgery, mornings, and afternoons of postoperative days 1 and 2	Sham: needles placed away from meridians Control: analgesic drug regimen	NR	NRS, analgesics, cardiovascular parameters, complications

Marra, 2011, Italy [54]	Pilot RCT	Randomized = 42 Completed = 42 Intervention: 21, >18 Control: 21, >18	Mediolateral episiotomy	Manual acupuncture	Lower 1 acupoint according to wrist-ankle acupuncture, right ankle Duration: needle left in place from discharge on day 2 or 3 after delivery, removed by midwife	Standard treatment without acupuncture	NR	Oral analgesics, perineal pain, acupuncture acceptability, AE

Wu, 2009, China [55]	RCT, three groups	Randomized = 60 Completed = 60 PCA only: 20, 30.8 (3.2) PCA + acupuncture: 20, 31.0 (4.2) PCA + EA: 20, 30.1 (4.1)	CS	Manual acupuncture, EA	SP6 Duration: 30 min, prior to PCA	No special treatment, PCA machine applied	NR	VAS, PCA demand, AE

Sertel, 2009, Germany [56]	Single-blind, randomized, prospective bicenter study	Randomized = 123 Completed = 123 Verum acupuncture: 41, 29.51 (16–58) Control acupuncture: 41, 26.6 (16–50) Medication: 41, 28.21 (16–58)	Tonsillectomy	Manual acupuncture	S34, S44, PC5 Duration: 20 min	Control acupuncture: nonspecific points away from meridians Medication: standard pain medication	NR	VAS, AE

Tsang, 2007, Hong Kong [57]	Prospective patient- and assessor- blinded RCT	Randomized = 36 Completed = 30 Intervention group: 15, 70.6 (5.8) Sham group: 15, 66.1 (7.5)	Bilateral total knee arthroplasty	Manual acupuncture	ST32, ST33, GB31, GB35, GB34, ST36 Duration: 20 min, with needle manipulation every 5 min to achieve numbness	No needle manipulation, no inducement of or inquiry about numbness, tingling, or heaviness	NR	VAS, analgesics, ROM, timed up-and-go test

Kotani, 2001, Japan [58]	RCT	*Upper abdominal surgery* Randomized = 107 Completed = 98 Intervention group: 50, 52 (15) Control group: 48, 55 (14) *Lower abdominal surgery* Randomized = 84 Completed = 77 Intervention group: 39, 55 (10) Control group: 38, 55 (11)	Abdominal surgery	Manual acupuncture	Upper abdominal surgery: bilateral BL18–BL24 Lower abdominal surgery: bilateral BL20–BL26 Duration: NR	Needles positioned at acupoints but not inserted into intradermal space	NR	VRS, analgesics, analgesic-related effects, PONV, plasma cortisol level, epinephrine requirement

Gupta, 1999, UK [59]	Prospective, double-blind, randomized controlled study	Randomized = 42 Completed = 42 Intervention group: 21, 40.1 (19.4) Control group: 21, 47.4 (19.6)	Elective single-knee arthroscopy	Manual acupuncture	SP9, SP10, ST34, ST36s Duration: 15 min, with manual stimulation for 5 s by 180° needle rotation every 5 min, and just before needle removal	Standard treatment	NR	VAS, analgesic requirement

Lao, 1999, USA [60]	Randomized, double-blind, placebo- controlled trial	Randomized = 39 Completed = 39 Intervention group: 19, 18–34 Control group: 20, 18–34	Oral surgery	Manual acupuncture	LI4, ST6, ST7, SJ17 Duration: 20 min, with manual manipulation for 20–30 s immediately after insertion, at midpoint, and at end of treatment	Identical to intervention treatment but no needle insertion into skin	7 days	Medication consumption, pain, psychological impact, AE

Lao, 1995, USA [61]	Randomized, single-blind, placebo- controlled trial	Randomized = 22, (18–40) Completed = 19 Intervention group: 11 Placebo acupuncture group: 8	Oral surgery	Manual acupuncture	LI4, ST6, ST7, SJ17s Duration: 20 min, with manual acupuncture on initially and at midpoint of treatment	Tapping next to acupoints to produce discernible sensation	7 days	Pain, medication consumption, AE, local discomfort

RCT, randomized controlled trial; APS, acupoint stimulation; EA, electroacupuncture; LF, low frequency; HF, high frequency; TEAS, transcutaneous electrical acupoint stimulation; NR, not reported; VAS, visual analog scale; PCA, patient-controlled analgesia; TAES, transcutaneous acupoint electrical stimulation; PONV, postoperative nausea and vomiting; RSS, Ramsay Sedation Scale; TENS, transcutaneous electrical nerve stimulation; HSS, New York Hospital for Special Surgery score; ROM, range of motion; MMT, manual muscle test; AES, acupoint electrical stimulation; AE, adverse effects/side effects; BPI, Brief Pain Inventory; BP, blood pressure; HR, heart rate; BCS, Bruggemann Comfort Scale; SF-36, Short Form 36; CS, cesarean section; PPR, percentile pain reduction; FVC, forced vital capacity; NRS, numerical rating scale; VRS, verbal rating scale; DDQ, Demographic Data Questionnaire; RINV, Rhodes Index of Nausea and Vomiting; VASA, Visual Analog Scale for Anxiety; STAI, State-Trait Anxiety Inventory.

**Table 3 tab3:** Characteristics of RCTs examining auricular acupoint stimulation included in the meta-analysis.

First author, year, setting	Study design	Participants (*n*), age (years)	Surgery type	APS type	Intervention		Follow-up	Main outcomes
					Intervention group	Sham/control group		
Zhang, 2013, China [62]	RCT	Randomized = 120 Completed = 120 Intervention group: 60, 35 (7) Placebo group: 60, 34 (8)	Gynecological laparoscopy	Seed embedding	TF4, CO4, and AH6A auricular points Duration: pressed for 5 min preoperatively and 1, 5, 9, and 23 h postoperatively	No sticking or pressing of vaccaria seeds	NR	PONV, VAS, tropisetron and morphine usage rates, adverse effects

He, 2013, China [24]	Prospective, randomized, sham-controlled trial	Randomized = 90 Completed = 90 Intervention group: 45, 61.58 (6.66) Control group: 45, 65.62 (6.10)	Total knee arthroplasty	Seed embedding	Site-knee joint, Shen Men, subcortex, and sympathesis auricular points Duration: pressed for 3 min four times per day until 7 days postoperatively	Sham acupuncture at four nonacupoints on helix ipsilateral to surgical site	3 months after operation	VAS, analgesic, analgesic-related adverse effects, HSS, ROM

Chang, 2012, Taiwan [6]	Double-blind RCT	Randomized = 62 Completed = 62 Intervention group: 31, 71.23 (7.09) Sham group: 31, 70.74 (8.09)	Total knee arthroplasty	Seed embedding	TF4 and AT4 auricular points Duration: pressed for 3 min three times per day (9 am, 1 pm, and 5 pm)	Regular care	NR	VAS, SF-MPQ, analgesic dosage, ROM

Kong, 2012, China [64]	RCT	Randomized = 60 Completed = 60 Intervention group: 30, 39.02 (11.19) Control group: 30, 38.17 (13.02)	Calcaneal fracture surgery	Seed embedding	Shen Men, thalamus lung, liver, kidney, and knee joint auricular points Duration: 10–15 min five times per day	Standard treatment (PCIA)	NR	VAS, analgesic, adverse reactions, urinary retention

Yeh, 2010, Taiwan [65]	Single-blind RCT	Randomized = 94 Completed = 74 Intervention group: 36, 58.8 (13.6) Control group: 38, 55.1 (16.1)	Lumbar spinal surgery	Seed embedding	TF4, AT3, AH9, CO4, CO3, and CO18 auricular points Duration: 3 minutes per point, four times per day until 72 h postoperatively	Standard treatment with no acupressure	NR	APSPOQ, pain intensity, analgesic dose, PONV, satisfaction

Tsang, 2011, Hong Kong [19]	Patient- and assessor-blinded RCT	Randomized = 48 Completed = 48 Intervention group: 16, 45.31 (2.68) Sham group: 16, 45.88 (3.91) Control group: 17, 44.63 (4.92)	Hysterectomy	EA	Uterus, abdomen, sympathetic, Shen Men, and subcortex auricular points Duration: several hours postoperatively	Sham TENS group: 90 s stimulation at 1 Hz Control group: 20 min bed rest	NR	VAS, PEFR

Kager, 2009, Austria [66]	Double-blind, randomized study	Randomized = 33 Completed = 33 Intervention group: 16, 30.78 (7.19) Placebo: 17, 30.92 (7.78)	Tonsillectomy	EA	Point 55 (entrance of the spirit, Shen Men), point 29 (occiput, pillow, Zhen), and point 73 (BIAN TAO Ti 1) Duration: 3 h application, 3 h pause for 96 h	Placement of acupuncture needles without stimulation, removed after 96 h	NR	VAS, NRS, oral analgesia, PONV

Michalek-Sauberer, 2007, Austria [67]	Prospective, randomized, double-blind, placebo- controlled study	Randomized = 149 Completed = 119 AA + AE group: 60, 27 (18–35) AA group: 41, 24 (20–35) Sham AE group: 41, 26 (19–35)	Unilateral mandibular third molar extraction	EA	AA points 1 (tooth), 55 (Shen Men), and 84 (mouth) Duration: 30 min prior to procedure, 3 h stimulation with 3 h pause	Sham AE with metal plates instead of needles, no electrical stimulation	NR	VAS, acetaminophen requirement, patient satisfaction

Sator-Katzenschlager, 2006, Austria [69]	Prospective, randomized, double-blind, controlled study	Randomized = 94 Completed = 93 EA group: 32, 33.3 (1.7) A group: 32, 34.2 (1.1) Control group: 30, 33.9 (1.9)	IVF treatment	EA	Auricular points 57 (uterus), 55 (Shen Men), and 29 (cushion) Duration: 30 min preoperatively until 1 h postoperatively	Adhesive tape over whole ear	NR	Side effects, satisfaction, IVF outcome data

Wang, 2012, China [63]	RCT	Randomized = 60 Completed = 60 Intervention group: 31, 60.19 (6.33) Control group: 29, 60.10 (6.10)	Total knee arthroplasty	Manual auriculotherapy	Shen Men, thalamus lung, and knee joint auricular points Duration: the night before surgery until the end of their surgeries.	Sham needle embedding at nonacupoints	NR	VAS, PONV, dizziness, drowsiness, PCA

Usichenko, 2007, Germany [68]	Prospective, patient- and evaluator-blinded, controlled study	Randomized = 120 Completed = 120 Intervention group: 61, 42.0 (14.6) Control group: 59, 43.8 (12.6)	Ambulatory knee surgery	Manual auriculotherapy	Knee joint, Shen Men, and lung auricular points Duration: preoperatively until first morning postoperatively	Invasive needle control at three nonacupoints of helix ipsilateral to surgical site	NR	VAS, analgesia, discharge time, nighttime sleep quality, adverse events

Usichenko, 2005, Germany [70, 71]	Prospective, patient- and evaluator-blinded, controlled pilot study	Randomized = 20 Completed = 18 Intervention group: 10, 33 (13) Control group: 8, 45 (15)	Ambulatory knee surgery	Manual auriculotherapy	Knee joint, Shen Men, and lung auricular points Duration: 5 min each before tracheal intubation, during most painful phase of surgery, and before extubation	Invasive needle control at nonacupoints of helix ipsilateral to surgical site	NR	VAS, analgesia requirement, discharge time, night sleep, adverse events

Usichenko, 2005, Germany [70, 71]	Prospective randomized patient-, anesthesiologist-, evaluator-, and analyst-blinded, sham-controlled study	Randomized = 61 Completed = 54 Intervention group: 29, 68 (10) Control group: 25, 66 (11)	Total hip arthroplasty	Manual auriculotherapy	Hip joint, Shen Men, lung, and thalamus auricular points Duration: AA needle retention up to 3 days postoperatively	Nonacupoints of helix ipsilateral to surgical site	NR	PCA demand, VAS, analgesia-related side effects

Li, 1994, China [72]	Double-blind, randomized study	Randomized = 48, 48.64 (11.35) Completed = 48 Chinese herb group: 16 Auricular acupoint group: 16 Epidural morphine group: 16	Surgery for liver cancer	Manual auriculotherapy	Chinese herb group: *Astragalus* L., *Angelica* L., *Ophiopogon* J., *Corydalis*, and Rhizome Pinelliae (10 mL, w/v = 1 g/mL) Duration: orally three times a day Auricular acupoint group: heart, lung, and Shen Men acupoints Duration: three times a day, 24 h postoperatively	Epidural morphine group: 2 mg epidural morphine before peritoneum suturing	NR	VAS, pethidine requirement, leucine enkephalin level

RCT, randomized controlled trial; APS, acupoint stimulation; NR, not reported; PONV, postoperative nausea and vomiting; VAS, visual analog scale; HSS, New York Hospital for Special Surgery score; ROM, range of motion; PCA, patient-controlled analgesia; SF-MPQ, Short-Form McGill Pain Questionnaire; PEFR, peak expiratory flow rate; APSPOQ, American Pain Society Patient Outcome Questionnaire; TENS, transcutaneous electrical nerve stimulation; NRS, numerical rating scale; AA/A, auricular acupuncture; AE/EA, auricular electroacupuncture; IVF, in vitro fertilization.

**Table 4:** Characteristics of RCTs examining integrative acupoint stimulation included in the meta-analysis.

First author, year, setting	Study design	Participants (*n*), age (years)	Surgery type	APS type	Intervention		Follow-up	Main outcomes
					Intervention group	Sham/control group		
Chung, 2014, Taiwan [24]	Single-blind, sham-controlled study, three groups	Enrolled = 135 Completed = 127 Intervention group: 40, 60.25 (13.42) Sham group: 42, 59.79 (12.61) Control group: 45, 54.24 (14.63)	Lumbar spinal surgery	AA + EA	TF4, AH10, CW8, AT5, CO4; BL40 and GB34 acupoints Duration: AA: ten 15 min sessions (1 and 3 h after returning to ward, four each on postoperative days 1 and 2) TAES: 20 min 1 and 3 h after returning to ward	Sham group: sham acupressure and sham TAES Control group: no acupoint stimulation	NR	VAS, morphine consumption, morphine-related side effects

Dias, 2010, Brazil [73]	Single-blind RCT	Randomized = 33 Completed = 33 Intervention group: 16, 47 (14) Control group: 17, 43.1 (10)	Inguinal hernia repair	EA	Acupoints on limbs and ear (heart and Shen Men) Duration: 5 min at 3 Hz in continuous mode, increased incrementally to 5, 10, 20, 50, 100, 160, and finally 240 Hz after 30 min	Sham TENS at same sites	2 weeks	Anesthetics requirement, VAS, HR, BP, edema, adverse events

Pfister, 2010, USA [74]	Prospective, open-label RCT	Randomized = 70 Completed = 58 Intervention group: 28, 61 Control group: 30, 57	Neck dissection	Manual acupuncture	LI4, SP6, GV20, Luo Zhen, and auricular Shen Men acupoints Duration: 30 min once a week for 4 weeks	Usual care	NR	NRS, medication use, adverse events

Deng, 2008, USA [75]	Randomized, sham-controlled, subject-blinded trial	Randomized = 162 Completed = 106 Intervention group: 52, 65 Sham group: 54, 63	Thoracotomy	Manual acupuncture	BL12–BL19, Wei Guan Xia Shu, ST36, and bilateral auricular Shen Men acupoints Duration: 4 weeks	Dummy studs placed in back	3 months postoperatively	BPI, medication use, MQS, NRS

Mehling, 2007, USA [76]	RCT	Randomized = 138 Completed = 138 Intervention group: 93, 55.9 (1.9) Control group: 45, 59.2 (1.7)	Cancer-related surgery	Massage + EA	Acupressure-type foot massage, large intestine 4, spleen 6, and auricular acupoints Duration: 10–30 min massage, depending on participants' clinical needs and conditions; 20 min acupuncture	Usual care, offered 30 min massage	NR	NRS, nausea, vomiting, POMS-SF, health care cost

Tavares, 2007, Brazil [77]	RCT	Randomized = 24, 20.42 (1.44) Completed = 24 Intervention group: 12 Control group: 12	Mandibular third molar surgery	EA	IG4, F3, E44, VB39, TA21, B60, Shen Men, and ponto total acupoints Duration: needles rotation every 10 min at beginning of procedure, after 10 min of treatment, and at end of procedure	No acupoint stimulation	NR	VAS, analgesic dose

Ekblom, 1991, Sweden [78]	RCT	Randomized = 110 Completed = 110 PRE-ACU group: 25 POST-ACU group: 25 Age, groups 1 and 2: 29.9 (18–50) Control group: 60, 30.2 (18–55)	Mandibular third molar extraction	Manual acupuncture	ST6, ST7, S119, and LI4 acupoints ipsilateral to extraction site; SJ5 contralateral Duration: 20 min	No acupuncture	1 week postoperatively	VAS, tension and stress, analgesic consumption, wound healing

RCT, randomized controlled trial; APS, acupoint stimulation; AA, auricular acupuncture; EA, electroacupuncture; TAES, transcutaneous acupoint electrical stimulation; NR, not reported; VAS, visual analog scale; TENS, transcutaneous electrical nerve stimulation; BP, blood pressure; HR, heart rate; NRS, numerical rating scale; BPI, Brief Pain Inventory; MQS, Medication Quantification Scale; POMS-SF, Mood States Short Form; PRE-ACU, preoperative acupuncture; POST-ACU, postoperative acupuncture.

**Table 5:** Summary of meta-analysis results.

Group	Outcome	Number of trials	Number of participants	Statistical method	Effect size	*P*	Heterogeneity^*∗*^
Overall effects (AM)							
I versus ST	VAS	20	1227	Std. MD (IV, random, 95% CI)	−1.05 (−1.44, −0.67)	<0.00001	*P* < 0.00001; *I* ^2^ = 89%
I versus S/P	VAS	23	1284	Std. MD (IV, random, 95% CI)	−0.72 (−1.03, −0.41)	<0.00001	*P* < 0.00001; *I* ^2^ = 85%
Body points (AM)	VAS	24	1370	Std. MD (IV, random, 95% CI)	−0.97 (−1.32, −0.62)	<0.00001	*P* < 0.00001; *I* ^2^ = 89%
I versus ST	VAS	14	893	Std. MD (IV, random, 95% CI)	−1.08 (−1.54, −0.61)	<0.00001	*P* < 0.00001; *I* ^2^ = 90%
I versus S/P	VAS	14	693	Std. MD (IV, random, 95% CI)	−0.86 (−1.28, −0.45)	<0.0001	*P* < 0.00001; *I* ^2^ = 84%
Body points (EA)	VAS	17	928	Std. MD (IV, random, 95% CI)	−0.71 (−1.02, −0.40)	<0.00001	*P* < 0.00001; *I* ^2^ = 80%
I versus ST	VAS	10	591	Std. MD (IV, random, 95% CI)	−0.68 (−1.04, −0.32)	0.0002	*P* < 0.00001; *I* ^2^ = 78%
I versus S/P	VAS	9	417	Std. MD (IV, random, 95% CI)	−0.67 (−1.13, −0.22)	0.004	*P* < 0.00001; *I* ^2^ = 80%
Body points (EA)-NIN	VAS	8	463	Std. MD (IV, random, 95% CI)	−0.66 (−1.10, −0.22)	0.003	*P* < 0.00001; *I* ^2^ = 80%
Body points (EA)-IN	VAS	10	545	Std. MD (IV, random, 95% CI)	−0.67 (−1.11, −0.24)	0.002	*P* < 0.00001; *I* ^2^ = 83%
Body points (M)	VAS	3	152	Std. MD (IV, random, 95% CI)	−1.55 (−3.91, 0.81)	0.20	*P* < 0.00001; *I* ^2^ = 97%
I versus ST	VAS	2	122	Std. MD (IV, random, 95% CI)	−2.43 (−5.49, 0.46)	0.12	*P* < 0.00001; *I* ^2^ = 97%
Body points (A)	VAS	3	214	MD (IV, random, 95% CI)	−1.44 (−2.56, −0.33)	0.01	*P* < 0.00001; *I* ^2^ = 92%
Body points (Pla) I versus ST	VAS	2	116	MD (IV, random, 95% CI)	−10.85 (−15.59, −6.12)	<0.00001	*P* = 0.09; *I* ^2^ = 66%
Body points for AS	VAS	8	471	Std. MD (IV, random, 95% CI)	−0.67 (−1.06, −0.29)	0.0006	*P* = 0.0001; *I* ^2^ = 76%
Body points for KS	VAS	4	200	Std. MD (IV, random, 95% CI)	−0.90 (−2.14, 0.34)	0.16	*P* < 0.00001; *I* ^2^ = 94%
Body points for CaS	VAS	2	52	Std. MD (IV, random, 95% CI)	−0.94 (−1.52, −0.36)	0.002	*P* = 0.98; *I* ^2^ = 0%
Body points for CeS	VAS	2	144	Std. MD (IV, random, 95% CI)	−0.81 (−1.15, −0.47)	<0.00001	*P* = 0.83; *I* ^2^ = 0%
I versus S/P	VAS	2	116	MD (IV, random, 95% CI)	−8.38 (−12.10, −4.65)	<0.0001	*P* = 0.13; *I* ^2^ = 56%
Auricular points (AM)	VAS	12	784	Std. MD (IV, random, 95% CI)	−0.66 (−1.06, −0.25)	0.001	*P* < 0.00001; *I* ^2^ = 86%
I versus ST	VAS	4	225	Std. MD (IV, random, 95% CI)	−1.23 (−2.44, −0.03)	0.04	*P* < 0.00001; *I* ^2^ = 92%
I versus S/P	VAS	9	591	Std. MD (IV, random, 95% CI)	−0.56 (−1.05, −0.07)	0.02	*P* < 0.00001; *I* ^2^ = 87%
Auricular points (EM)	VAS	5	404	Std. MD (IV, random, 95% CI)	−0.77 (−1.42, −0.12)	0.02	*P* < 0.00001; *I* ^2^ = 90%
Auricular points (M)	VAS	4	252	Std. MD (IV, random, 95% CI)	−0.16 (−0.62, 0.30)	0.49	*P* = 0.04; *I* ^2^ = 64%
Auricular points (EA)	VAS	3	128	Std. MD (IV, random, 95% CI)	−1.11 (−1.60, −0.61)	<0.0001	*P* = 0.19; *I* ^2^ = 40%
Auricular points for KS	VAS	5	350	Std. MD (IV, random, 95% CI)	−0.27 (−0.68, 0.14)	0.20	*P* = 0.009; *I* ^2^ = 70%
Auricular points for AS	VAS	2	138	Std. MD (IV, random, 95% CI)	−1.19 (−2.12, −0.26)	0.01	*P* = 0.07; *I* ^2^ = 70%
Integrative points	VAS/NRS	6	444	Std. MD (IV, random, 95% CI)	−0.61 (−1.14, −0.07)	0.03	*P* < 0.0001; *I* ^2^ = 84%
Integrative points for OS	VAS	2	109	Std. MD (IV, random, 95% CI)	−1.87 (−5.69, 1.94)	0.34	*P* < 0.00001; *I* ^2^ = 96%
Analgesic requirement	TMC	6	399	MD (IV, random, 95% CI)	−4.99 (−7.51, −2.47)	0.0001	*P* < 0.00001; *I* ^2^ = 94%
I versus S/P	TMC	2	132	MD (IV, random, 95% CI)	−15.20 (−21.24, −9.16)	<0.00001	*P* = 1; *I* ^2^ = 0%

AM, all modalities; VAS, visual analog scale; NRS, numerical rating scale; I, intervention group; ST, standard treatment control group; S/P, sham/placebo control group; EA, electroacupuncture; NIN, noninvasive; IN, invasive; M, manual acupuncture; A, acupressure; Pla, plaster; EM, seed embedding; TMC: total morphine consumption; Std. MD, Std. mean difference; MD, mean difference; AS, abdominal surgery; KS, knee surgery; CaS, cardiac surgery; CeS, cesarean section; OS: oral surgery.

^*∗*^ *χ* ^2^ test.

**Table 6:** Sensitivity analysis results.

Presensitivity analysis statistical method	Heterogeneity	Sensitivity analysis	Number of trials (patient *N*)	Postsensitivity analysis statistical method	Heterogeneity
Effects of interventions, all body modalities versus standard treatment control (VAS)					
Random, −1.08 (−1.54, −0.61)	*P* < 0.00001; *I* ^2^ = 90%	Remove Kim et al. 2009 [23] and Sertel et al. 2009 [56]	12 (*755*)	Random, −0.74 (−1.05, −0.43)	*P* < 0.00001; *I* ^2^ = 76%

Effects of invasive body electroacupuncture versus all controls (VAS)					
Random, −0.67 (−1.11, −0.24)	*P* < 0.00001; *I* ^2^ = 83%	Remove Gilbertson et al. 2003 [41]	9 (*505*)	Random, −0.47 (−0.79, −0.15)	*P* = 0.002; *I* ^2^ = 68%

Effects of body electroacupuncture versus standard treatment (VAS)					
Random, −0.68 (−1.04, −0.32)	*P* < 0.00001; *I* ^2^ = 78%	Remove Deng et al. 2010 [35] and Chen et al. 2012 [32]	8 (*461*)	Fixed, −0.38 (−0.57, −0.20)	*P* = 0.33; *I* ^2^ = 13%

Effects of body electroacupuncture versus sham/placebo control (VAS)					
Random, −0.67 (−1.13, −0.22)	*P* < 0.00001; *I* ^2^ = 80%	Remove Gilbertson et al. 2003 [41]	8 (*377*)	Fixed, −0.42 (−0.62, −0.21)	*P* = 0.08; *I* ^2^ = 44%

Effects of body acupressure versus standard treatment (VAS)					
Random, −1.44 (−2.56, −0.33)	*P* < 0.00001; *I* ^2^ = 92%	Remove Adib-Hajbaghery and Etri 2013 [51]	2 (*144*)	Fixed, −1.93 (−2.33, −1.53)	*P* = 0.75; *I* ^2^ = 0%

Overall effects of auricular interventions, all modalities versus sham/placebo control (VAS)					
Random, −0.56 (−1.05, −0.07)	*P* < 0.00001; *I* ^2^ = 87%	Remove Zhang 2013 [62] and Sator-Katzenschlager et al. 2006 [69]	7 (*409*)	Random, −0.23 (−0.54, 0.09)	*P* = 0.04; *I* ^2^ = 55%

Effects of auricular manual acupuncture versus sham/placebo control (VAS)					
Random, −0.16 (−0.62, 0.30)	*P* = 0.04; *I* ^2^ = 64%	Remove Usichenko et al. 2007 [68]	3 (*132*)	Fixed, −0.34 (−0.69, 0.00)	*P* = 0.25; *I* ^2^ = 27%

Overall effects of integrative interventions versus all controls (VAS and NRS)					
Random, −0.61 (−1.14, −0.07)	*P* < 0.0001; *I* ^2^ = 84%	Remove Tavares et al. 2007 [77]	5 (*420*)	Fixed, −0.29 (−0.49, −0.09)	*P* = 0.12; *I* ^2^ = 46%

## Appendix

### A. Searching Strategies

#### A.1. PubMed

#1 “acupuncture”[MeSH Terms] OR “acupuncture therapy”[MeSH Terms] OR “acupuncture analgesia”[MeSH Terms] OR “acupuncture points”[MeSH Terms] OR “acupressure”[MeSH Terms] OR “auriculotherapy”[MeSH Terms] OR “acupuncture, ear”[MeSH Terms]
#2 ((((((((((((((acupunctur ^*∗*^[Title/Abstract]) OR acupoin ^*∗*^[Title/Abstract]) OR acupressur ^*∗*^[Title/Abstract]) OR auriculotherap ^*∗*^[Title/Abstract]) OR (auricu ^*∗*^[Title/Abstract] AND poin ^*∗*^[Title/Abstract])) OR (ear[Title/Abstract] AND poin ^*∗*^[Title/Abstract])) OR (auricu ^*∗*^[Title/Abstract] AND acupoin ^*∗*^[Title/Abstract])) OR (ear[Title/Abstract] AND acupoin ^*∗*^[Title/Abstract])) OR (auricu ^*∗*^[Title/Abstract] AND plaster ^*∗*^[Title/Abstract])) OR (massag ^*∗*^[Title/Abstract] AND ear[Title/Abstract])) OR (ear[Title/Abstract] AND plaster ^*∗*^[Title/Abstract])) OR (massag ^*∗*^[Title/Abstract] AND auricu ^*∗*^[Title/Abstract])) OR (magnetic[Title/Abstract] AND ear[Title/Abstract])) OR (magnetic[Title/Abstract] AND auricu ^*∗*^[Title/Abstract])) OR otopoin ^*∗*^[Title/Abstract] OR vaccaria ^*∗*^[Title/Abstract]
#3 #1 OR #2
#4 “perioperative period”[MeSH Terms] OR “postoperative period”[MeSH Terms] OR “preoperative period”[MeSH Terms] OR “intraoperative period”[MeSH Terms]
#5 (((((perioperati ^*∗*^[Title/Abstract]) OR surger ^*∗*^[Title/Abstract]) OR preoperati ^*∗*^[Title/Abstract]) OR intraoperati ^*∗*^) OR postoperati ^*∗*^[Title/Abstract]) OR operati ^*∗*^
#6 #4 OR #5
#7 ((((pain[Title/Abstract]) OR ache[Title/Abstract])) OR (“pain”[MeSH Terms] OR “acute pain”[MeSH Terms] OR “pain management”[MeSH Terms] OR “chronic pain”[MeSH Terms]) OR “analgesia”[MeSH Terms])
#8 #3 AND #6 AND #7
#9 ((((((((“randomized controlled trial”[Publication Type]) OR “controlled clinical trial”[Publication Type]) OR “ramdomized”[Title/Abstract]) OR “ramdomised”[Title/Abstract]) OR “placebo”[Title/Abstract]) OR “sham”[Title/Abstract]) OR “randomly”[Title/Abstract]) OR “trial”[Title/Abstract]) OR “groups”[Title/Abstract]
#10 (animals[MeSH Terms] NOT (humans[MeSH Terms] AND animals[MeSH Terms]))
#11 #9 NOT #10
#12 #8 AND #11

#### A.2. Embase

#1 ‘acupuncture'/exp
#2 ‘acupuncture analgesia'/exp
#3 ‘acupressure'/exp
#4 acupunctur ^*∗*^:ab,ti OR acupoin ^*∗*^:ab,ti OR acupressur ^*∗*^:ab,ti OR auriculotherap ^*∗*^:ab,ti OR (auricu ^*∗*^ NEAR/3 acupunctur ^*∗*^):ab,ti OR (auricu ^*∗*^ NEAR/3 acupressur ^*∗*^):ab,ti OR (auricu ^*∗*^ NEAR/3 poin ^*∗*^):ab,ti OR ‘auricular plaster':ab,ti OR (ear NEAR/3 plaster ^*∗*^):ab,ti OR (ear NEAR/3 poin ^*∗*^):ab,ti OR (ear NEAR/3 acupoint ^*∗*^):ab,ti OR otopoin ^*∗*^:ab,ti OR earhole ^*∗*^:ab,ti OR (vaccaria ^*∗*^ NEAR/15 ear):ab,ti OR (vaccaria ^*∗*^ NEAR/15 auricu ^*∗*^):ab,ti OR (massag ^*∗*^ NEAR/3 auricu ^*∗*^):ab,ti OR (massag ^*∗*^ NEAR/3 ear):ab,ti OR (cowherb NEAR/15 ear):ab,ti OR (cowherb NEAR/15 auricu ^*∗*^):ab,ti OR (seed ^*∗*^ NEAR/15 auricu ^*∗*^):ab,ti OR (seed ^*∗*^ NEAR/15 ear):ab,ti OR (magnetic NEAR/15 ear):ab,ti OR (magnetic NEAR/15 auricu ^*∗*^):ab,ti OR erxue ^*∗*^:ab,ti
#5 #1 OR #2 OR #3 OR #4
#6 ‘perioperative period'/exp
#7 ‘postoperative period'/exp
#8 ‘preoperative period'/exp
#9 ‘intraoperative period'/exp
#10 ‘surgery'/exp
#11 ‘perioperative period':ab,ti OR operative:ab,ti OR surgery:ab,ti OR (peri NEAR/3 operative):ab,ti OR (post ^*∗*^ NEAR/5 operative):ab,ti OR (pre ^*∗*^ NEAR/5 operative):ab,ti OR (intra ^*∗*^ NEAR/5 operative):ab,ti
#12 #6 OR #7 OR #8 OR #9 OR #10 OR #11
#13 ‘pain'/exp
#14 ‘analgesia'/exp
#15 pain ^*∗*^:ab,ti OR analgesia:ab,ti OR ache ^*∗*^:ab,ti OR (pain NEAR/3 management):ab,ti OR (pain NEAR/3 control):ab,ti
#16 #13 OR #14 OR #15
#17 #5 AND #12 AND #16
#18 ‘controlled clinical trial'/exp OR ‘single blind procedure'/exp OR ‘double-blind procedure'/exp OR ‘crossover procedure'/exp
#19 random ^*∗*^:ab,ti OR crossover ^*∗*^:ab,ti OR (cross NEAR/3 over ^*∗*^):ab,ti OR placebo:ab,ti OR (doubl ^*∗*^ NEAR/3 blind ^*∗*^):ab,ti OR (doubl ^*∗*^ NEAR/3 mask ^*∗*^):ab,ti OR (singl ^*∗*^ NEAR/3 blind ^*∗*^):ab,ti OR (singl ^*∗*^ NEAR/3 mask ^*∗*^):ab,ti OR (trebl ^*∗*^ NEAR/3 blind ^*∗*^):ab,ti OR (trebl ^*∗*^ NEAR/3 mask ^*∗*^):ab,ti OR (tripl ^*∗*^ NEAR/3 blind ^*∗*^):ab,ti OR (tripl ^*∗*^ NEAR/3 mask ^*∗*^):ab,ti OR assign ^*∗*^:ab,ti OR allocat ^*∗*^:ab,ti OR volunteer ^*∗*^:ab,ti
#20 #18 OR #19
#21 ‘animal'/exp OR ‘nonhuman'/exp OR ‘animal experiment'/exp
#22 ‘human'/exp
#23 #21 AND #22
#24 #21 NOT #23
#25 #20 NOT #24
#26 #17 AND #25

#### A.3. Cochrane Central Register of Controlled Trials (CENTRAL)

#1 MeSH descriptor: [Acupuncture] explode all trees
#2 MeSH descriptor: [Auriculotherapy] explode all trees
#3 MeSH descriptor: [Acupressure] explode all trees
#4 MeSH descriptor: [Acupuncture Analgesia] explode all trees
#5 MeSH descriptor: [Acupuncture, Ear] explode all trees
#6 acupunctur ^*∗*^ or acupressur ^*∗*^ or acupoin ^*∗*^ or auriculotherap ^*∗*^ or (auricu ^*∗*^ near/3 poin ^*∗*^) or (ear near/3 poin ^*∗*^) or (ear near/3 plaster ^*∗*^) or (auricu ^*∗*^ near/3 plaster ^*∗*^) or (ear near/3 acupoint ^*∗*^) or otopoint ^*∗*^ or earhole ^*∗*^ or (vaccaria ^*∗*^ near/15 ear) or (vaccaria ^*∗*^ near/15 auricu ^*∗*^) or (cowherb near/15 ear) or (cowherb near/15 auricu ^*∗*^) or (magnetic near/15 ear) or (magnetic near/15 auricu ^*∗*^) or (massag ^*∗*^ near/3 ear) or (massag ^*∗*^ near/3 auricu ^*∗*^) or erxue ^*∗*^:ti,ab,kw (Word variations have been searched)
#7 #1 OR #2 OR #3 OR #4 OR #5 OR #6
#8 MeSH descriptor: [General Surgery] explode all trees
#9 perioperative period or operati ^*∗*^ or surger ^*∗*^ or (peri ^*∗*^ near/5 operati ^*∗*^) or (post ^*∗*^ near/5 operati ^*∗*^) or (pre ^*∗*^ near/5 operati ^*∗*^) or (intra ^*∗*^ near/5 operati ^*∗*^):ti,ab,kw (Word variations have been searched)
#10 #8 OR #9
#11 MeSH descriptor: [Pain] explode all trees
#12 MeSH descriptor: [Analgesia] explode all trees
#13 pain ^*∗*^ or analgesia or ache ^*∗*^ or (pain near/3 management) OR (pain near/3 control):ti,ab,kw (Word variations have been searched)
#14 #11 OR #12 OR #13
#15 #7 AND #10 AND #14
#16 #15 in Trials

#### A.4. Cumulative Index to Nursing and Allied Health Literature (CINAHL)

#1 MM Acupuncture OR MM Auriculotherapy OR MM Acupuncture, Ear OR MM acupressure OR MM acupuncture analgesia OR MM acupuncture points
#2 TI acupunctur ^*∗*^ OR acupoin ^*∗*^ OR acupressur ^*∗*^ OR auriculotherap ^*∗*^ OR (auricu ^*∗*^ N3 acupunctur ^*∗*^) OR (ear N3 poin ^*∗*^) OR (ear N3 plaster ^*∗*^) OR (auricu ^*∗*^ N3 plaster ^*∗*^) OR (ear N5 acupoint ^*∗*^) OR otopoint ^*∗*^ OR earhole ^*∗*^ OR (vaccaria ^*∗*^ N15 ear) OR (vaccaria ^*∗*^ N15 auricu ^*∗*^) OR (magnetic N15 ear) OR (magne ^*∗*^ N15 auricu ^*∗*^) OR (massag^*∗*^ N3 ear) OR (massag ^*∗*^ N3 auricu ^*∗*^)
#3 AB acupunctur ^*∗*^ OR acupoin ^*∗*^ OR acupressur ^*∗*^ OR auriculotherap ^*∗*^ OR (auricu ^*∗*^ N3 acupunctur ^*∗*^) OR (ear N3 poin ^*∗*^) OR (ear N3 plaster ^*∗*^) OR (auricu ^*∗*^ N3 plaster ^*∗*^) OR (ear N5 acupoint ^*∗*^) OR otopoint ^*∗*^ OR earhole ^*∗*^ OR (vaccaria ^*∗*^ N15 ear) OR (vaccaria ^*∗*^ N15 auricu ^*∗*^) OR (magnetic N15 ear) OR (magne ^*∗*^ N15 auricu ^*∗*^) OR (massag ^*∗*^ N3 ear) OR (massag ^*∗*^ N3 auricu ^*∗*^)
#4 #1 OR #2 OR #3
#5 MM perioperative period OR MM postoperative period OR MM preoperative period OR MM intraoperative period
#6 TI perioperati ^*∗*^ OR surger ^*∗*^ OR preoperati ^*∗*^ OR intraoperati ^*∗*^ OR postoperati ^*∗*^ OR operati ^*∗*^ OR (peri ^*∗*^ N5 operati ^*∗*^) OR (post ^*∗*^ N5 operati ^*∗*^) OR (pre ^*∗*^ N5 operati ^*∗*^) OR (intra ^*∗*^ N5 operati ^*∗*^)
#7 AB perioperati ^*∗*^ OR surger ^*∗*^ OR preoperati ^*∗*^ OR intraoperati ^*∗*^ OR postoperati ^*∗*^ OR operati ^*∗*^ OR (peri ^*∗*^ N5 operati ^*∗*^) OR (post ^*∗*^ N5 operati ^*∗*^) OR (pre ^*∗*^ N5 operati ^*∗*^) OR (intra ^*∗*^ N5 operati ^*∗*^)
#8 #5 OR #6 OR #7
#9 MM Pain OR MM Analgesia
#10 TI pain ^*∗*^ OR ache ^*∗*^ OR analgesia ^*∗*^ OR (pain ^*∗*^ N5 management ^*∗*^) OR (pian ^*∗*^ N5 contral ^*∗*^)
#11 AB pain^*∗*^ OR ache^*∗*^ OR analgesia ^*∗*^ OR (pain ^*∗*^ N5 management ^*∗*^) OR (pian ^*∗*^ N5 contral^*∗*^)
#12 #9 OR #10 OR #11
#13 #4 AND #8 AND #12

#### A.5. PsycINFO

#1 MJSUB.EXACT.EXPLODE(“Acupuncture”)
#2 TI(acupunctur ^*∗*^ OR acupressur ^*∗*^ OR acupoin ^*∗*^ OR auriculotherap ^*∗*^ OR auricu ^*∗*^ NEAR/3 acupunctur ^*∗*^ OR auricu ^*∗*^ NEAR/3 poin ^*∗*^ OR ear NEAR/3 plaster ^*∗*^ OR auricu ^*∗*^ NEAR/3 plaster ^*∗*^ OR ear NEAR/3 poin ^*∗*^ OR ear NEAR/3 acupoint ^*∗*^ OR otopoint ^*∗*^ OR earhole ^*∗*^ OR vaccaria ^*∗*^ NEAR/15 ear OR vaccaria ^*∗*^ NEAR/15 auricu ^*∗*^ OR cowherb NEAR/15 ear OR cowherb NEAR/15 auricu ^*∗*^ OR magne ^*∗*^ NEAR/15 ear OR magne ^*∗*^ NEAR/15 auricu ^*∗*^ OR massag ^*∗*^ NEAR/3 ear OR erxue ^*∗*^ OR massag ^*∗*^ NEAR/3 auricu ^*∗*^)
#3 AB(acupunctur ^*∗*^ OR acupressur ^*∗*^ OR acupoin ^*∗*^ OR auriculotherap ^*∗*^ OR auricu ^*∗*^ NEAR/3 acupunctur ^*∗*^ OR auricu ^*∗*^ NEAR/3 poin ^*∗*^ OR ear NEAR/3 plaster ^*∗*^ OR auricu ^*∗*^ NEAR/3 plaster ^*∗*^ OR ear NEAR/3 poin ^*∗*^ OR ear NEAR/3 acupoint ^*∗*^ OR otopoint ^*∗*^ OR earhole ^*∗*^ OR vaccaria ^*∗*^ NEAR/15 ear OR vaccaria ^*∗*^ NEAR/15 auricu ^*∗*^ OR cowherb NEAR/15 ear OR cowherb NEAR/15 auricu ^*∗*^ OR magne ^*∗*^ NEAR/15 ear OR magne ^*∗*^ NEAR/15 auricu ^*∗*^ OR massag ^*∗*^ NEAR/3 ear OR erxue ^*∗*^ OR massag ^*∗*^ NEAR/3 auricu ^*∗*^)
#4 #1 OR #2 OR #3
#5 MJSUB.EXACT.EXPLODE(“surgery”)
#6 TI(perioperati ^*∗*^ OR surger ^*∗*^ OR preoperati ^*∗*^ OR intraoperati ^*∗*^ OR postoperati ^*∗*^ OR operati ^*∗*^)
#7 AB(perioperati ^*∗*^ OR surger ^*∗*^ OR preoperati ^*∗*^ OR intraoperati ^*∗*^ OR postoperati ^*∗*^ OR operati ^*∗*^)
#8 #5 OR #6 OR #7
#9 MJSUB.EXACT.EXPLODE(“pain”)
#10 TI(pain ^*∗*^ OR ache ^*∗*^ OR analgesia ^*∗*^)
#11 AB(pain ^*∗*^ OR ache ^*∗*^ OR analgesia ^*∗*^)
#12 #9 OR #10 OR #11
#13 #4 AND #8 AND #12
#14 TI(random ^*∗*^ OR crossover ^*∗*^ OR cross NEAR/3 over ^*∗*^ OR placebo OR double ^*∗*^ NEAR/3 blind ^*∗*^ OR double ^*∗*^ NEAR/3 mask ^*∗*^ OR single ^*∗*^ NEAR/3 blind ^*∗*^ OR single ^*∗*^ NEAR/3 mask ^*∗*^ OR treble ^*∗*^ NEAR/3 blind ^*∗*^ OR treble ^*∗*^ NEAR/3 mask ^*∗*^ OR triple ^*∗*^ NEAR/3 blind ^*∗*^ OR triple ^*∗*^ NEAR/3 mask ^*∗*^ OR assign ^*∗*^ OR allocate ^*∗*^ OR volunteer ^*∗*^)
#15 AB(random ^*∗*^ OR crossover ^*∗*^ OR cross NEAR/3 over ^*∗*^ OR placebo OR double ^*∗*^ NEAR/3 blind ^*∗*^ OR double ^*∗*^ NEAR/3 mask ^*∗*^ OR single ^*∗*^ NEAR/3 blind ^*∗*^ OR single ^*∗*^ NEAR/3 mask ^*∗*^ OR treble ^*∗*^ NEAR/3 blind ^*∗*^ OR treble ^*∗*^ NEAR/3 mask ^*∗*^ OR triple ^*∗*^ NEAR/3 blind ^*∗*^OR triple ^*∗*^ NEAR/3 mask ^*∗*^ OR assign ^*∗*^ OR allocate ^*∗*^ OR volunteer ^*∗*^)
#16 #14 OR #15
#17 #13 AND #16

#### A.6. Allied and Complementary Medicine (AMED)

#1 exp acupuncture/
#2 exp acupressure/
#3 exp Ear acupuncture/
#4 (“acupunctur ^*∗*^” or “acupoin ^*∗*^” or “acupressur ^*∗*^” or “auriculotherap ^*∗*^” or “ear adj3 acupressur ^*∗*^” or “auricu ^*∗*^ adj3 acupressur ^*∗*^” or “ear adj3 plaster ^*∗*^” or “ear adj3 acupoint ^*∗*^” or “otopoin ^*∗*^” or “earhole ^*∗*^” or “vaccaria ^*∗*^ adj15 ear” or “vaccaria ^*∗*^ adj15 auricu ^*∗*^” or “massag ^*∗*^ adj3 auricu ^*∗*^” or “massag ^*∗*^ adj3 ear” or “cowherb adj15 auricu ^*∗*^” or “seed ^*∗*^ adj15 auricu ^*∗*^” or “seed ^*∗*^ adj15 ear” or “magnetic adj15 auricu ^*∗*^” or “erxue ^*∗*^”).ti.
#5 (“acupunctur ^*∗*^” or “acupoin ^*∗*^” or “acupressur ^*∗*^” or “auriculotherap ^*∗*^” or “ear adj3 acupressur ^*∗*^” or “auricu ^*∗*^ adj3 acupressur ^*∗*^” or “ear adj3 plaster ^*∗*^” or “ear adj3 acupoint ^*∗*^” or “otopoin ^*∗*^” or “earhole ^*∗*^” or “vaccaria ^*∗*^ adj15 ear” or “vaccaria ^*∗*^ adj15 auricu ^*∗*^” or “massag ^*∗*^ adj3 auricu ^*∗*^” or “massag ^*∗*^ adj3 ear” or “cowherb adj15 auricu ^*∗*^” or “seed ^*∗*^ adj15 auricu ^*∗*^” or “seed ^*∗*^ adj15 ear” or “magnetic adj15 auricu ^*∗*^” or “erxue ^*∗*^”).ab.
#6 #1 OR #2 OR #3 OR #4 OR #5
#7 exp surgery/
#8 (“perioperative period” or “postoperative period” or “preoperative period” or “intraoperative period” or “perioperati ^*∗*^” or “surger ^*∗*^” or “preoperati ^*∗*^” or “intraoperati ^*∗*^” or “postoperati ^*∗*^” or “operati ^*∗*^”).ti.
#9 (“perioperative period” or “postoperative period” or “preoperative period” or “intraoperative period” or “perioperati ^*∗*^” or “surger ^*∗*^” or “preoperati ^*∗*^” or “intraoperati ^*∗*^” or “postoperati ^*∗*^” or “operati ^*∗*^”).ab.
#10 #7 OR #8 OR #9
#11 exp pain/
#12 (“pain ^*∗*^” or “ache ^*∗*^” or “pain adj3 management ^*∗*^” or “pain adj3 control” or “analgesia”).ti.
#13 (“pain ^*∗*^” or “ache ^*∗*^” or “pain adj3 management ^*∗*^” or “pain adj3 control” or “analgesia”).ab.
#14 #11 OR #12 OR #13
#15 #6 AND #10 AND #14
#16 exp Random allocation/ or exp Clinical trials/ or exp Randomized controlled trials/ or exp Placebos/
#17 (“random ^*∗*^” or “crossover ^*∗*^” or “cross adj3 over ^*∗*^” or “placebo” or “doubl ^*∗*^ adj3 blind ^*∗*^” or “doubl ^*∗*^ adj3 mask ^*∗*^” or “singl ^*∗*^ adj3 blind ^*∗*^” or “singl ^*∗*^ adj3 mask ^*∗*^” or “trebl ^*∗*^ adj3 blind ^*∗*^” or “trebl ^*∗*^ adj3 mask ^*∗*^” or “tripl ^*∗*^ adj3 blind ^*∗*^” or “allocat ^*∗*^” or “ volunteer ^*∗*^” or “tripl ^*∗*^ adj3 mask ^*∗*^” or “assign ^*∗*^”).ti.
#18 (“random ^*∗*^” or “crossover ^*∗*^” or “cross adj3 over ^*∗*^” or “placebo” or “doubl ^*∗*^ adj3 blind ^*∗*^” or “doubl ^*∗*^ adj3 mask ^*∗*^” or “singl ^*∗*^ adj3 blind ^*∗*^” or “singl ^*∗*^ adj3 mask ^*∗*^” or “trebl ^*∗*^ adj3 blind ^*∗*^” or “trebl ^*∗*^ adj3 mask ^*∗*^” or “tripl ^*∗*^ adj3 blind ^*∗*^” or “allocat ^*∗*^” or “volunteer ^*∗*^” or “tripl ^*∗*^ adj3 mask ^*∗*^” or “assign ^*∗*^”).ab.
#19 #16 OR #17 OR #18
#20 #15 AND #19

#### A.7. Thomson Reuters Web of Science

#1 TS = (acupunctur ^*∗*^ OR acupoin ^*∗*^ OR acupressur ^*∗*^ OR auriculotherap ^*∗*^ OR (auricu ^*∗*^ NEAR/3 acupunctur ^*∗*^) OR (auricu ^*∗*^ NEAR/3 poin ^*∗*^) OR (ear NEAR/3 plaster ^*∗*^) OR (auricu ^*∗*^ NEAR/3 plaster ^*∗*^) OR (ear NEAR/3 poin ^*∗*^) OR (auricu ^*∗*^ NEAR/3 acupoint ^*∗*^) OR (ear NEAR/3 acupoint ^*∗*^) OR otopoint ^*∗*^ OR (vaccaria ^*∗*^ NEAR/15 ear) OR (vaccaria ^*∗*^ NEAR/15 auricu ^*∗*^) OR (cowherb NEAR/15 auricu ^*∗*^) OR (magnetic NEAR/15 ear) OR (magnetic NEAR/15 auricu ^*∗*^) OR (massag ^*∗*^ NEAR/3 ear) OR (massag ^*∗*^ NEAR/3 auricu ^*∗*^) OR erxue ^*∗*^) Indexes = SCI-EXPANDED, SSCI, A&HCI, CPCI-S, CPCI-SSH Timespan = All years
#2 TS = (perioperative period OR postoperative period OR preoperative period OR intraoperative period OR perioperati ^*∗*^ OR surger ^*∗*^ OR preoperati ^*∗*^ OR intraoperati ^*∗*^ OR postoperati ^*∗*^ OR operati ^*∗*^ OR (peri ^*∗*^ NEAR/3 operati ^*∗*^) OR (post ^*∗*^ NEAR/3 operati ^*∗*^) OR (pre NEAR/3 operati ^*∗*^) OR intra ^*∗*^ NEAR/3 operati ^*∗*^) Indexes = SCI-EXPANDED, SSCI, A&HCI, CPCI-S, CPCI-SSH Timespan = All years
#3 TS = ((acute NEAR/3 pain ^*∗*^) OR (chronic NEAR/3 pain ^*∗*^) OR (pain NEAR/3 management ^*∗*^) OR (pain NEAR/3 control ^*∗*^) OR pain ^*∗*^ OR ache OR analgesia ^*∗*^) Indexes = SCI-EXPANDED, SSCI, A&HCI, CPCI-S, CPCI-SSH Timespan = All years
#4 #1 AND #2 AND #3

#### A.8. ScienceDirect

#1 TITLE-ABSTR-KEY(acupuncture OR acupoint OR acupressure OR auriculotherapy OR (ear W/5 acupressur ^*∗*^) OR (auricu ^*∗*^ W/5 acupressur ^*∗*^) OR (auricu ^*∗*^ W/5 poin ^*∗*^) OR (vaccaria ^*∗*^ W/5 auricu ^*∗*^) OR (cowherb W/5 auricu ^*∗*^) OR (magnetic W/5 auricu ^*∗*^) OR (massag ^*∗*^ W/5 auricu ^*∗*^) OR erxue ^*∗*^) [All Sources(- All Sciences -)]
#2 TITLE-ABSTR-KEY(perioperative period OR postoperative period OR preoperative period OR intraoperative period OR perioperati ^*∗*^ OR surger ^*∗*^ OR preoperati ^*∗*^ OR intraoperati ^*∗*^ OR postoperati ^*∗*^ OR operati ^*∗*^)[All Sources(- All Sciences -)]
#3 TITLE-ABSTR-KEY(pain ^*∗*^ OR ache ^*∗*^ OR acute pain OR chronic pain OR (pain ^*∗*^ W/5 management ^*∗*^) OR (pain ^*∗*^ W/5 control ^*∗*^) OR analgesia) [All Sources(- All Sciences -)]
#4 #1 AND #2 AND #3
